# Mesenchymal stromal cells alleviate acute respiratory distress syndrome through the cholinergic anti-inflammatory pathway

**DOI:** 10.1038/s41392-022-01124-6

**Published:** 2022-09-05

**Authors:** Xiaoran Zhang, Xuxia Wei, Yiwen Deng, Xiaofeng Yuan, Jiahao Shi, Weijun Huang, Jing Huang, Xiaoyong Chen, Shuwei Zheng, Jieying Chen, Keyu Chen, Ruiming Xu, Hongmiao Wang, Weiqiang Li, Shiyue Li, Huimin Yi, Andy Peng Xiang

**Affiliations:** 1 Department of Surgical Intensive Care, The Third Affiliated Hospital, Sun Yat-Sen University, Guangzhou, Guangdong 510630 China; 2 Center for Stem Cell Biology and Tissue Engineering, Key Laboratory for Stem Cells and Tissue Engineering, Ministry of Education, Sun Yat-Sen University, Guangzhou, Guangdong 510080 China; 3 National-Local Joint Engineering Research Center for Stem Cells and Regenerative Medicine, Zhongshan School of Medicine, Sun Yat-Sen University, Guangzhou, Guangdong 510080 China; 4 Department of General Intensive Care Unit, Lingnan Hospital, The Third Affiliated Hospital of Sun Yat-sen University, Guangzhou, Guangdong 510530 China; 5 Department of Anesthesiology, The First Affiliated Hospital, Sun Yat-sen University, Guangzhou, Guangdong 510080 China; 6 State Key Laboratory of Respiratory Disease and National Clinical Research Center for Respiratory Disease, the First Affiliated Hospital of Guangzhou Medical University, Guangzhou Medical University, Guangzhou, Guangdong 510120 China

**Keywords:** Mesenchymal stem cells, Respiratory tract diseases

## Abstract

Mesenchymal stromal cells (MSCs) have been considered a promising alternative for treatment of acute respiratory distress syndrome (ARDS). However, there is significant heterogeneity in their therapeutic efficacy, largely owing to the incomplete understanding of the mechanisms underlying the therapeutic activities of MSCs. Here, we hypothesize that the cholinergic anti-inflammatory pathway (CAP), which is recognized as a neuroimmunological pathway, may be involved in the therapeutic mechanisms by which MSCs mitigate ARDS. Using lipopolysaccharide (LPS) and bacterial lung inflammation models, we found that inflammatory cell infiltration and Evans blue leakage were reduced and that the expression levels of choline acetyltransferase (ChAT) and vesicular acetylcholine transporter (VAChT) in lung tissue were significantly increased 6 hours after MSC infusion. When the vagus nerve was blocked or α7 nicotinic acetylcholine (ACh) receptor (α7nAChR)-knockout mice were used, the therapeutic effects of MSCs were significantly reduced, suggesting that the CAP may play an important role in the effects of MSCs in ARDS treatment. Our results further showed that MSC-derived prostaglandin E2 (PGE2) likely promoted ACh synthesis and release. Additionally, based on the efficacy of nAChR and α7nAChR agonists, we found that lobeline, the nicotinic cholinergic receptor excitation stimulant, may attenuate pulmonary inflammation and alleviate respiratory symptoms of ARDS patients in a clinical study (ChiCTR2100047403). In summary, we reveal a previously unrecognized MSC-mediated mechanism of CAP activation as the means by which MSCs alleviate ARDS-like syndrome, providing insight into the clinical translation of MSCs or CAP-related strategies for the treatment of patients with ARDS.

## Introduction

Acute respiratory distress syndrome (ARDS) is a potentially reversible condition with mortality rates as high as 30% to 40%. It is clinically defined as respiratory failure that occurs within 1 week of an insult or new/worsening respiratory symptoms, and is accompanied by bilateral opacities on chest radiographs.^1,2^ As ARDS progresses, alveolar macrophages defend against pathogens and trigger inflammatory responses in the distal respiratory tract.^3^ Hallmarks of this disease include uncontrolled alveolar inflammation as well as overproduction of pro-inflammatory mediators (hyperinflammatory status), which contribute to lung injury and respiratory failure.^4^ Despite improvements in supportive care and ventilator management, ARDS continues to be associated with high mortality and morbidity rates.^5,6^ Additionally, the COVID-19 pandemic is rapidly and continuously spreading worldwide, causing severe respiratory illness and death. The most frequently documented reason that patients require intensive care is the need for respiratory support, and two-thirds of these patients meet the criteria for ARDS.^7^ As a consequence, there is a tremendous need to investigate efficacious therapeutic strategies for ARDS.

As a potential treatment for ARDS, mesenchymal stromal cells (MSCs) are attractive due to their the pleiotropic properties.^8^ MSCs are multipotent cells that possess low immunogenicity, releasing a number of paracrine factors, including anti-inflammatory cytokines, anti-apoptosis factors, and antibacterial peptides.^9–11^ Several preclinical studies have shown that MSCs are effective for alleviating the severity of acute lung injury.^12,13^ Our single-armed clinical study suggested that a single bolus dose of MSCs was safe in 22 patients with persistent ARDS and significantly alleviated lung injury; in these patients, pulse oxygen saturation% (SpO2) significantly increased within the first 12 hours posttreatment, and the bronchoalveolar lavage fluid (BALF) levels of TNF-α and IL-6 showed sustained improvement after infusion.^14^ Similarly, in a phase 1 clinical trial, moderate or severe ARDS patients tolerated the MSC treatment well and showed improved lung injury scores, as well as decreased IL-6 and IL-8 levels.^15^ However, MSCs failed to improve clinical symptoms in a randomized phase 2a clinical trial.^16^ These results indicate there is an urgent need for elucidating the action of mechanisms by which MSCs exert therapeutic effects in patients with ARDS.

The autonomic innervation of the human airways contributes to many aspects of airway function. Compared with the sparse distribution of sympathetic or adrenergic nervous system, parasympathetic nerves are the dominant neural components that control airway smooth muscle tone and airway secretions.^17,18^ Moreover, the parasympathetic nerve systems can release the neurotransmitter acetylcholine (ACh) to control a neuroimmunomodulatory pathway called the cholinergic anti-inflammatory pathway (CAP) that may inhibit overproduction of local cytokine and prevent the damaging effects.^19,20^ Tracey KJ et al. discovered that activation of this pathway may help inhibit cytokine release locally in tissues, without causing systemic immunosuppression, and alleviate severe sepsis.^20–22^ In addition, we and others reported that MSCs first resided in the lung after infusion and then migrate to the parenchyma and airways.^23,24^ Therefore, we hypothesized that the therapeutic effects of MSCs in lung injury might occur via the CAP.

Here, we reveal that MSC treatment significantly protects mice against bacterial pneumonia or LPS-induced lung injury via the CAP pathway. When the CAP was inhibited through vagotomy (VGX) and pharmacological and genetic ablation experiments, the anti-inflammatory effects of MSCs were markedly reduced in lung injury models.

## Results

### MSC treatment improves lung injury via the CAP

To examine the anti-inflammatory effects of MSCs on the lungs, we established a model of ARDS by inducing lung injury via the administration of lipopolysaccharide (LPS) to C57Bl/6 J mice by nasal drip. A total of 1 × 10^6^ human dermal fibroblasts (HDFs) or MSCs were intravenously administered 4 hours after injury. Histological examination and lung injury scores, which were scored according to American Thoracic Society work report method,^25^ showed that compared with the phosphate-buffered saline (PBS) control, MSC treatment greatly reduced the destruction of alveoli within 6 hours (Supplementary Fig. 1a, b), reduced Evans blue accumulation in the lung parenchyma, and decreased the levels of infiltrating cells in the BALF; however, HDF treatment did not improve the symptoms of lung injury (Supplementary Fig. 1c–e). These results suggest that MSCs, but not HDFs, alleviated the symptoms on lung injury in this model.

**Fig. 1:**
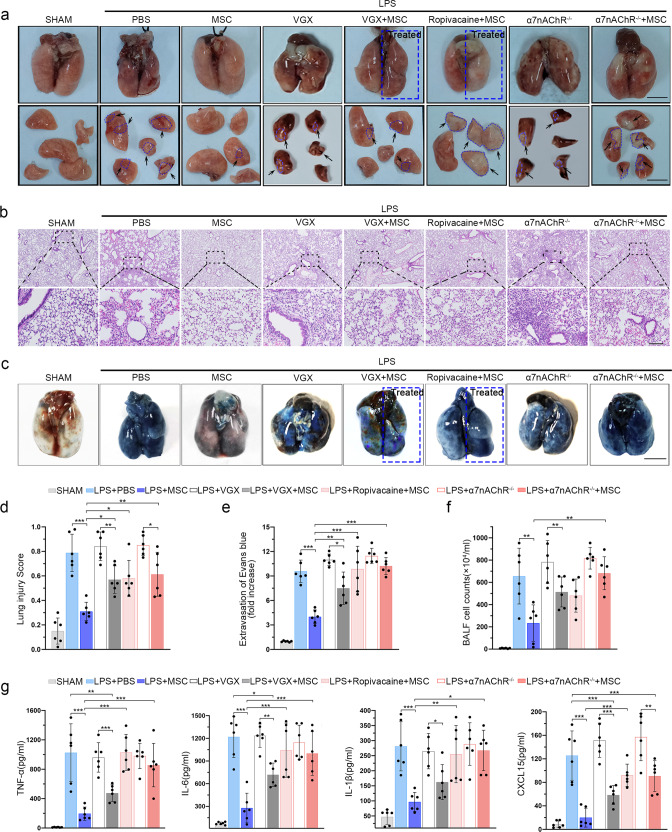
MSC treatment improves lung injury via the CAP. **a** Representative images of lung tissues were displayed from all groups. Scale bars, 4 cm. **b** Representative H&E staining was performed on lung samples from each group. Photographs of at least 6 sections of each tissue sample were taken. Scale bars, 100 μm. **c** Photographs showing Evans blue accumulation in lung tissues. **d** Quantitative analysis of the lung injury score; *n* = 6. **e** Evans blue extracted from lung tissues were quantified by spectrophotometric analysis; *n* = 6. **f** BALF total cell numbers were quantified; *n* = 6. **g** CXCL15, IL-1β, TNF-α, IL-6 ELISA kits were used to determine BALF inflammatory concentrations; *n* = 6. *p < 0.05, **p < 0.01, ***p < 0.001

To investigate whether the CAP is involved in the ability of MSCs to mitigate ARDS, we performed several inhibition experiments, including VGX and vagal anesthesia (Supplementary Fig. 1f). Macroscopic view and histological examination of the lungs of the LPS-treated mice revealed widespread septal thickening and evident interstitial immune cell infiltration. As expected, MSC infusion resulted in a significant reduction in lung inflammation (Fig. 1a–f). After VGX, the mice treated with LPS exhibited serious lung injury, and MSCs reduced the consolidation in the segments or lobes of the lungs in some degree, but not as obviously as LPS + MSC group (Fig. 1a–e). To further confirm this observation, we used another approach, namely, anesthetizing the right side of the vagus nerve, to block the CAP. Interestingly, in the ropivacaine-treated group, the therapeutic effect of MSCs was observed only in contralateral lung tissues, whereas prominent lung consolidation was observed in the anesthetized side (Fig. 1a). MSC-treatment also failed to exert the desired effects on the histological results (Fig. 1b, d). MSCs were not able to ameliorate lung vascular permeability in the ropivacaine-treated side (Fig. 1c, e), and Evans blue staining showed that extravasation into the lung tissues of MSC-infused mice was ten times higher in the anesthetized side than in the nonanesthetized side (Supplementary Fig. 1g). In addition, a previous study demonstrated that activation of α7nAChR could inhibit inflammatory cytokine release via the CAP pathway.^26^ Here, we used LPS to induce lung injury in α7nAChR^−/−^ mice, and MSC treatment failed to ameliorate Evans blue accumulation and the number of infiltrating cells in the BALF (Fig. 1a–f). Prevention of a cytokine storm, in particular, decreasing TNF-α, IL-6, IL-1β, and CXCL15 production, is a key strategy for the treatment of ARDS. As shown in Fig. 1g, MSCs notably inhibited the LPS-induced increases in the production of these cytokines. When the vagus nerve was blocked, these anti-inflammatory effects of MSCs disappear, especially in LPS + Ropivacaine+MSC and LPS + α7nAChR^−/−^+MSC group. We further established the bacterial pneumonia model (intratracheal injection with *Staphylococcus aureus* (*S. aureus*) or *Escherichia coli* (*E. coli*)) to measure the therapeutic effects of MSCs and the critical role of the CAP pathway. MSC treatment significantly alleviated the symptoms of lung injury, as significantly increasing the survival rates, decreasing cell counts and the bacterial load in the BALF (Supplementary Fig. 2a–e). Moreover, MSCs failed to ameliorate bacterial pneumonia in α7nAChR^−/−^ mice (Supplementary Fig. 2f–j). Our results suggest that MSC infusion significantly improve the symptoms of acute lung injury induced by LPS or bacterial pneumonia partially through the CAP pathway.

### MSCs facilitate the upregulation of ChAT and VAChT expression in injured lungs

**Fig. 2:**
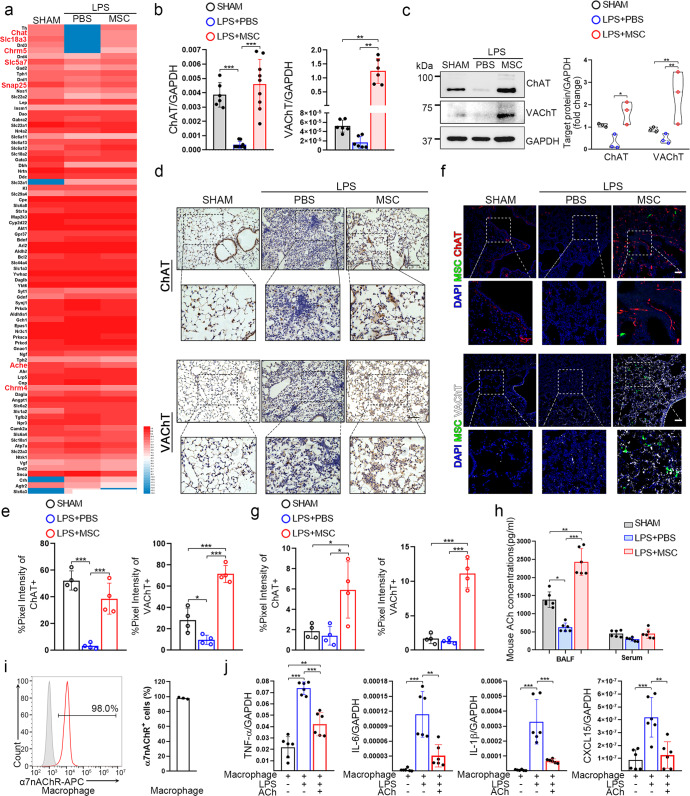
MSCs facilitate the upregulation of choline ChAT and VAChT expression in injured lungs. **a** Heatmap showing neurotransmitter synthesis/transport-related genes expression levels in each group. Genes were ranked according to fold changes in expression between the PBS and MSC treatment group. The expression values of the genes were normalized to the Z score before the heatmap was generated; *n* = 3. Relative mRNA expression (**b**) and protein levels (**c**) of ChAT and VAChT in MSCs from each group. Representative immunohistochemical staining (**d**) and quantification (**e**) of ChAT (left) or VAChT (right) pixel intensity; scale bars, 100 μm; *n* = 4. Representative confocal images (**f**) of lung sections from different groups stained for MSCs (green) and ChAT (red; upper) or VAChT (white; lower). DAPI staining showed the cell nuclei (blue). Scale bars, 100 μm. Quantification (**g**) of the percentage of ChAT (left) or VAChT (right) pixel intensity; *n* = 4. **h** LC-MS/MS was used to detect BALF and serum ACh concentrations; *n* = 6. **i** Representative plots of α7nAChR expression by macrophages; *n* = 3. **j** Relative mRNA expression of CXCL15, TNF-α, IL-1β, and IL-6 in macrophages; LPS (10 μg/ml) and/or ACh (1 mM) were used to stimulate macrophages. *n* = 6

To further investigate the effectiveness of MSCs on CAP activation, the lung tissues before and after MSC treatment were collected and performed bulk sequencing. MSCs significantly increased the expression of ChAT, VAChT, and other cholinergic system-related genes (Fig. 2a). The data has been deposited in the national genomics data center (accession no. CRA007353). These results were verified by qRT–PCR and Western blotting analyses of homogenized lung tissues from the indicated groups (Fig. 2b, c). Immunohistochemical staining revealed strikingly decreased expression levels of ChAT and VAChT after injury, and MSC administration significantly restored the expression levels of these molecules (Fig. 2d, e). To further verify the expression of ChAT, we crossed knock-in ChAT Cre/+ mice with tdTomato reporter mice that express red fluorescence protein (RFP) after Cre-mediated recombination and observed similar results (Fig. 2f, g). VAChT was also highly expressed in MSC-treated mice compared to sham- and PBS-treated mice (Fig. 2f, g). The ACh concentrations in the BALF and serum of the MSC-treated group were notably higher than those in the other groups (Fig. 2h). Similarly, the expression of ChAT and VAChT, and the ACh concentration of the MSC-treated group were also notably higher than those of *S. aureus* and *E. coli* group (Supplementary Fig. 3a–e). Collectively, these results show that MSCs could promote ACh synthesis and release by upregulating the expressions of ChAT and VAChT.

### ACh inhibits proinflammatory cytokine production and mitigates the macrophage inflammatory response

BALF macrophages are critical initiator in increased vascular permeability and early neutrophil infiltration, and inhibition of macrophage activation may reduce inflammatory infiltration and lung injury.^5,27^ To determine the direct effect of ACh on macrophages, which expressed α7nAChR (Fig. 2i), we exposed LPS-stimulated macrophages to ACh in vitro and observed decreased expression levels of inflammatory factors (Fig. 2j). Previous reports showed that activation of α7nAChR on macrophages decreased the release of proinflammatory cytokines by inhibiting the activation of p65, which translocates to the nucleus upon IκBα degradation^28^ and induce the signal transducer and activator of transcription 3 (STAT3), phosphorylation of Janus kinase 2 (JAK2).^28^ We observed increased levels of phosphorylated JAK2 and STAT3 in ACh-stimulated macrophages compared to LPS-treated macrophages, whereas the levels of p65 and phosphorylated IκBα were decreased in ACh-stimulated macrophages (Supplementary Fig. 4a, b). The results of flow analysis of phosphorylation further demonstrated that ACh could activate α7nAChR-mediated pathways in macrophages (Supplementary Fig. 4c, d). These results suggest that ACh could regulate CAP signaling in relation to the JAK2/STAT3 and NF-kB pathways in macrophages.

### MSCs activate the CAP partially through the COX-2/PGE2 pathway

**Fig. 3:**
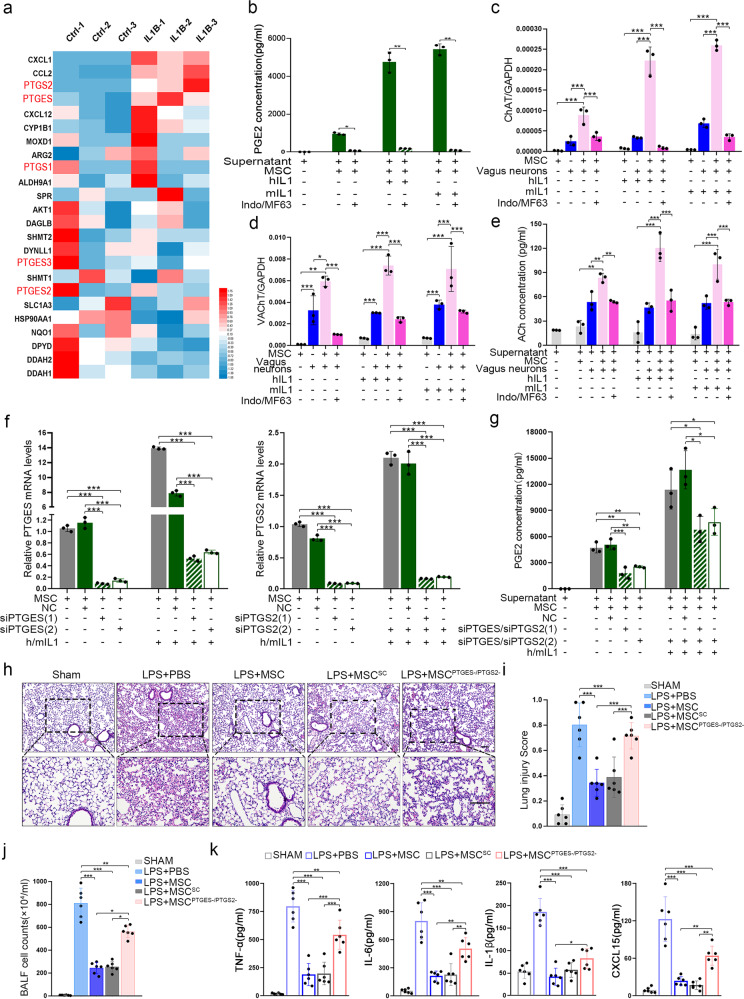
Prostaglandin E2 (PGE2) synthesis by MSCs triggers CAP-related effects. **a** RNA-seq data analysis was performed with MSCs isolated from 3 donors. The heatmap displays genes encoding neuromodulator or neuromodulator biosynthetic process-related proteins. The heatmap represents the fold changes in expression between unstimulated and IL-1β-stimulated MSCs. The expression levels of the neuromodulator/neuromodulator biosynthetic process-related genes were normalized to the Z score before the heatmap was generated; *n* = 3. **b** The concentrations of PGE2 in the supernatants of MSCs treated with or without hIL1, mhIL1, indomethacin, and MF63; *n* = 3. Relative mRNA expression levels of ChAT (**c**) and VAChT (**d**) in vagus neurons from each group; *n* = 3. **e** LC-MS/MS was used to detect ACh concentrations in the supernatants. *n* = 3. **f** Relative mRNA expression levels of PTGES and PTGS2 in MSCs from each group; *n* = 3. **g** PGE2 ELISA kit was used to detect the supernatant concentrations; *n* = 3. **h** Representative H&E staining was performed on lung samples from each group. Photographs of at least 6 sections of each tissue sample were taken. Scale bars, 100 μm. **i** Quantitative analysis of the lung injury score; *n* = 6. **j** BALF total cell numbers were quantified; *n* = 6. **k** CXCL15, IL-1β, TNF-α, IL-6 ELISA kits were used to determine BALF inflammatory concentrations; *n* = 6

To explore how MSCs activate the CAP, we examined the inflammatory factor expression profile and found that IL-1β was one of the most abundant and most highly upregulated factors in the lung injury model (Supplementary Fig. 5a). Genome-wide RNA sequencing (RNA-seq) was performed on MSCs before and after stimulated with IL-1β. The bullk RNA-seq data (accession no. SRP095307) were derived from our previous study.^29^ CXCL1, CCL2, prostaglandin-endoperoxide synthase 2 (PGES-2), and prostaglandin E synthase (PTGES) were highly expressed after stimulation. Because CXCL1 and CCL2 chemokines typically expressed by macrophages and neutrophils, they might not be candidate molecules for CAP activation^30^ (Fig. 3a). Prostaglandin E2 (PGE2), which is synthesized mainly by PTGS2 and PTGES, was previously reported to participate the regulation of ChAT expression.^31^ Here, we found that both human and murine IL-1β (hIL-1β, mIL-1β) could increase PGE2 production by MSCs (Fig. 3b). Indomethacin and MF63, which inhibit the activities of PTGS2 and PTGES, respectively, suppressed the production of PGE2 by IL-1β-stimulated MSCs (Fig. 3b). To explore the role of PGE2 in the ability of MSCs to regulate ChAT and VAChT expression in vitro, we cocultured MSCs with neurons freshly isolated from the cervical vagus ganglion. ChAT and VAChT were expressed at markedly higher levels in MSC cocultures than that each individual culture, and PGE2 synthetase inhibitors reduced the expression of these factors (Fig. 3c, d). hIL-1β or mIL-1β substantially upregulated the expression of ChAT and VAChT in cocultures, and the expression of these molecules was reduced by PGE2 synthetase inhibitors (Fig. 3c, d). Measurement of the ACh concentrations in the supernatants revealed that ACh levels were increased in the coculture system and notably decreased by PTGS2 and PTGES inhibitors (Fig. 3e).

PTGS2 and PTGES were knocked down in MSCs (MSC^PTGS-/PTGES-^) to confirm their role in MSC-mediated regulation of the CAP. Compared with wild-type MSCs and those transfected with scramble siRNAs (MSC^SC^), MSC^PTGS-/PTGES-^ expressed lower levels of PTGS2 and PTGES in the absence or presence of hIL-1β/mIL-1β (Fig. 3f). The PGE2 levels in the supernatants were measured, and similar results were observed (Fig. 3g). In the lung injury mouse model, MSC^PTGS-/PTGES-^ could not ameliorate the disruption of the alveolar septa or the infiltration of inflammatory cells as effectively as MSCs or MSC^SC^ (Fig. 3h). A higher lung injury score and higher numbers of cells in the BALF were observed in the MSC^PTGS-/PTGES-^ treatment group than in the MSC or MSC^SC^ treatment groups (Fig. 3i, j). MSC^PTGS-/PTGES-^ showed a decreased ability to inhibit the expression of inflammatory cytokines (Fig. 3k). Together, these results suggest that MSCs activate the CAP by producing PGE2.

### Effects of α7nAChR agonists in treating LPS-induced lung injury

**Fig. 4:**
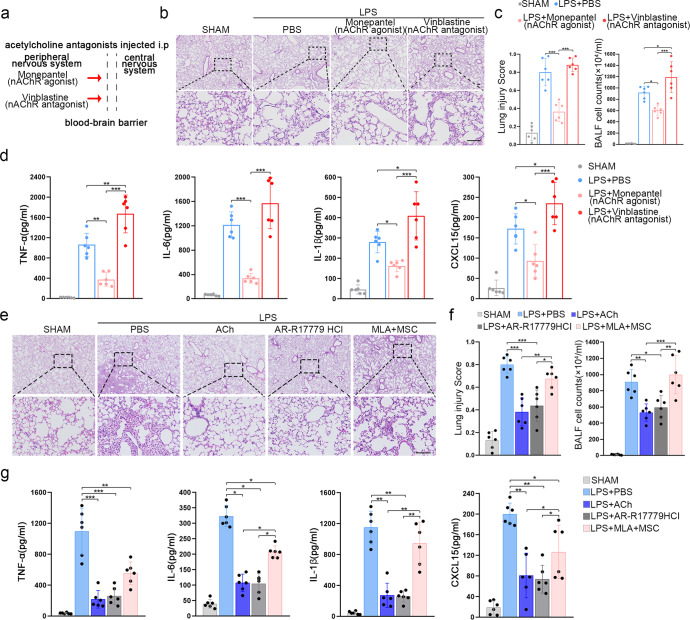
Effects of α7nAChR agonists in the treatment of LPS-induced lung injury. **a** The therapeutic effects of peripheral nAChR agonist monepantel and the peripheral nAChR antagonist vinblastine in acute lung injury were investigated. **b** Representative H&E staining was performed on lung samples from each group. Photographs of at least 6 sections of each tissue sample were taken. Scale bars, 100 μm. **c** The degree of lung injury was calculated and quantitatively analyzed according to the results of H&E staining, and the infiltration of leukocytes into BALF was compared among the groups; *n* = 6. **d** CXCL15, IL-1β, TNF-α, IL-6 ELISA kits were used to determine BALF inflammatory concentrations; *n* = 6. **e** Representative H&E staining was performed on lung samples from each group. Photographs of at least 6 sections of each tissue sample were taken. Scale bars, 100 μm. **f** The degree of lung injury was calculated and quantitatively analyzed according to the results of H&E staining, and the infiltration of leukocytes into BALF was compared among the groups; *n* = 6. **g** CXCL15, IL-1β, TNF-α, IL-6 ELISA kits were used to determine BALF inflammatory concentrations; *n* = 6

Our findings showed that MSCs promoted ACh synthesis and releasing by upregulating the ChAT and VAChT expressions. Because ACh performs its functions through nicotinic, muscarinic ACh receptors (nAChRs, mAChRs, respectively), we questioned whether the activation of one or more specific ACh receptors could exert therapeutic effects in the context of lung injury. Peripheral mAChR agonist (arecoline hydrobromide) or antagonist (methylbenactyzium bromide) treatment did not exert any apparent therapeutic effects (Supplementary Fig. 6a–d). The peripheral nAChR agonist monepantel significantly reduced lung injury, which was characterized by decreased infiltrating cells and inflammatory factors in BALF (Fig. 4a–d). Conversely, when nAChRs were blocked with the peripheral antagonist vinblastine (Fig. 4a), we observed a more severe inflammatory response than that observed in the PBS treatment group (Fig. 4b–d). Thus, nAChR agonists appear to be a potential treatment for LPS-induced lung injury. Among the nAChRs, α7nAChR has been associated with anti-inflammatory pathways.^32^ Consistent with the above findings, both specific α7nAChR agonists AR-R17779 hydrochloride (AR-R17779 HCl) and GTS-21 statistically inhibited inflammation and improved lung injury (Fig. 4e–g; Supplementary Fig. 6e–k). Conversely, specific blockade of α7nAChR with methyllycaconitine citrate (MLA) greatly reduced the therapeutic effects of MSCs (Fig. 4e–g). Together, these results indicate that nAChR and α7nAChR agonists notably inhibited proinflammatory cytokine release and exerted therapeutic effects on lung injury.

### The therapeutic effects of lobeline in treating lung injury

**Fig. 5:**
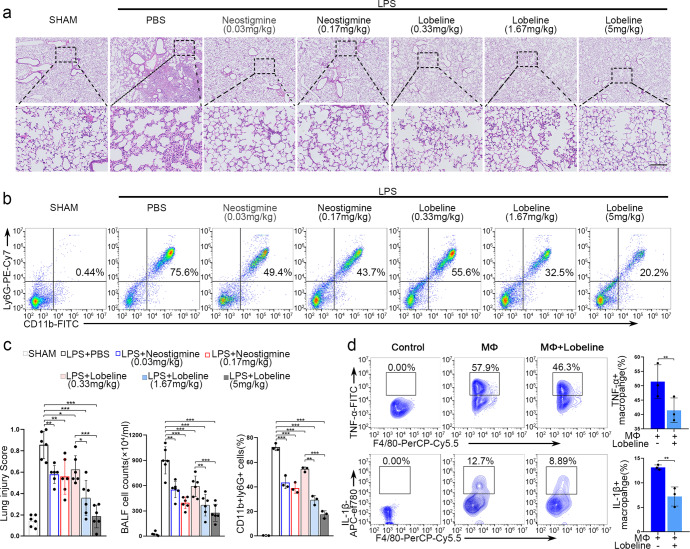
The potential anti-inflammatory properties of lobeline in the treatment of lung injury. **a** Representative H&E staining was performed on lung samples from each group. Photographs of at least 6 sections of each tissue sample were taken. Scale bars, 100 μm. **b** The numbers of BALF-infiltrating neutrophils in each group were analyzed by flow cytometric analysis. **c** The degree of lung injury was calculated and quantitatively analyzed according to the results of H&E staining, and the infiltration of leukocytes into BALF was compared among the groups; *n* = 6. Quantification of the percentage of neutrophils in BALF is shown; *n* = 3. **d** Representative plots of the percentage of TNF-α + and IL-1β + macrophages and quantification of the results were presented; *n* = 3

Considering that new synthesized drugs (the specific α7nAChR agonist, e.g. GTS-21) have not been approved immediately to patients, new use of conventional drug might be a feasible solution. We next explore the effect of cholinergic system-related drugs (parasympathetic stimulants) on murine ARDS and patients with ARDS. Both neostigmine (an anticholinesterase drug) and lobeline (a nicotinic cholinergic receptor activator) attenuated LPS-induced pulmonary inflammation (Fig. 5a). The number of intra-alveolar neutrophils are markers of the levels of cell infiltration into the BALF.^33^ The lung injury score, BALF cell count and proportion of CD11b + Ly6G+ neutrophils were significantly reduced by treatment with both neostigmine and lobeline (Fig. 5b, c). The therapeutic effects of lobeline appeared to be dose dependent, with a higher dose resulting in better efficacy (Fig. 5a–c). More importantly, lobeline exerted its effects over a wider range of doses than neostigmine, suggesting that lobeline might be safer, which is consistent with previous reports.^34^ Thus, we chose lobeline for our subsequent studies. Since macrophages in the alveoli acting an important source of inflammatory cytokines, the production of which can result in cytokine storms, we further explored whether lobeline could inhibit the production of inflammatory cytokines by macrophages in vitro. Indeed, lobeline notably suppressed the TNF-α and IL-1β releasing (two pivotal inflammatory mediators in LPS-induced lung injury) (Fig. 5d). Lobeline also inhibited the expression of TNF-α by T cells (Supplementary Fig. 7a). The results suggest that lobeline could be a potential candidate for treating ARDS by activating nAChRs.

### Lobeline improves the respiratory symptoms of ARDS patients

Given the efficacy observed in the mouse model of ARDS above, we postulated that lobeline treatment might be useful in patients with ARDS. Respiratory stimulation with lobeline has been used to treat respiratory depression due to various causes. To investigate the safety and efficacy of lobeline, we conducted a pilot, open-label, nonrandomized, controlled clinical trial (Table S1). With the approval of the ethical committee and the informed consents by the family members, according to the Berlin definition (with a sustained oxygenation index (OI) < 200 within 24 hours), 16 patients with moderate to severe ARDS were enrolled for treatment.^35^ Baseline clinical characteristics of recruited participants were basically the same between the control and treatment groups (Tables 1 and S2).

**Table 1:** Characteristics of the patients from control and lobeline groups

	Control group (*n* = 6)	Lobeline group (*n* = 10)
Age, years	59 (12)	60 (13)
Sex		
Female	2 (33.3%)	4 (40%)
Male	4 (66.7%)	6 (60%)
Sequential Organ Failure Assessment score	15.7 ± 3.09	12.9 ± 2.21
Time from ICU admission to ARDS diagnosis, days	2.91 (1.69)	3.5 (1.5)
Time from intubation to ARDS diagnosis, days	1.25 (0.56)	1.9 (0.83)
Cause of ARDS		
Pneumonia	2 (33%)	4 (40%)
Sepsis	1 (17%)	4 (40%)
Aspiration	0	1 (10%)
Trauma	1 (17%)	1 (10%)
Others	2 (33%)	0
Degree of Lung severity, number of patients		
Moderate (100 < PaO_2_/FiO_2_ ≤ 200)	5	9
Severe (PaO_2_/FiO_2_ ≤ 100)	1	1
PaO_2_/FiO_2_, mm Hg	133 (33.41)	143 (29.92)
Tidal volume, mL per predicted bodyweight	6.4 (0.32)	6.6 (0.48)
Respiratory rate, breaths per min	18.5 (1.38)	21.1 (6.39)
Positive end-expiratory pressure, cm H_2_O	10.2 (0.90)	11.1 (2.04)
Inspiratory plateau pressure, cm H_2_O	26.3 (1.11)	28.3 (3.19)

Data are *n* (%), mean (SD), unless otherwise stated

For the primary safety evaluation, vital signs were measured in Supplementary Fig. 8a. The heart rates, respiratory breathing rates, arterial pressures of the patients remained steady in both groups. With no relevant adverse events found, lobeline (20 mg administered via a slow intravenous drip for 1 day) was considered to be safe.

**Fig. 6:**
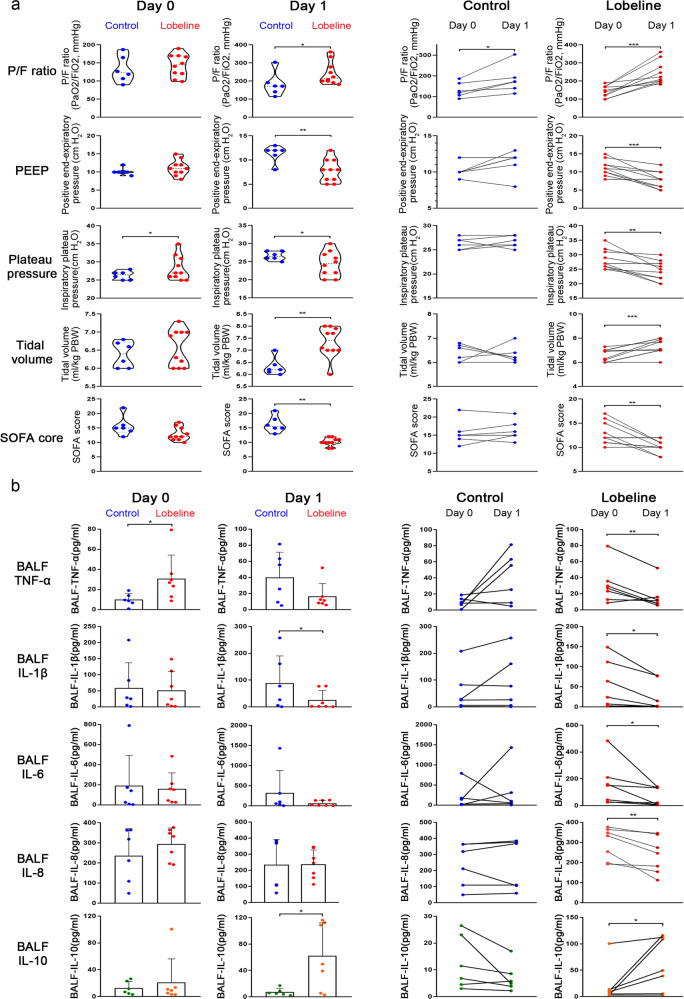
Lobeline improves the respiratory symptoms of ARDS patients. **a** Analysis of pulmonary ventilation index and multiple organ assessment. P/F ratio [PaO_2_(mmHg)/FiO_2_(%)], plateau pressure, positive end-expiratory pressure, tidal volume, and SOFA score were measured from day 0 to day 1 from two groups. **b** Analysis of inflammatory factors in BALF from two group patients. IL-8, IL-1β, TNF-α, IL-6, IFN-γ, IL-10 ELISA kits were used to determine BALF inflammatory concentrations

Primary efficacy outcome showed patients in the lobeline group had a greater mean number of ventilator-free days and higher ICU survival rate (Table 2). Pulmonary ventilation index, such as partial pressure of arterial oxygen (PaO_2_) to fraction of inspired oxygen (FiO_2_) ratio (P/F), positive end-expiratory airway pressure (PEEP), plateau pressure, and tidal volume were assessed. Baseline levels of these indicators were comparable in both groups. At 1 day after treatment initiation, we observed that P/F and tidal volume values were significantly higher in lobeline group, and PEEP and plateau pressure were notably decreased (Fig. 6a). We observed that significant improvement of ventilation index only in the lobeline treated patients, and multiple organ assessment respiratory mechanics SOFA score was markedly decreased in lobeline group (Fig. 6a).

**Table 2:** Main clinical outcomes

	Control group (*n* = 6)	Lobeline group (*n* = 10)	Between-group difference (95% CI)	*p* value
Ventilator-free days at 28 days	2.83 (3.04)	8.3 (9.06)	−5.47% (−10.58 to −0.36)	0.037^a^
ICU survival rate	1 (16.67%)	8 (80%)	−0.63% (−0.25 to −1.02)	0.035^a^
Hospital survival rate	1 (16.67%)	4 (40%)	−0.24% (−0.66 to 0.19)	0.588

Data are *n* (%), mean (SD), or median with between-group difference (95% CI). To compare categorical variables, Fisher’s exact test was used

*ICU* intensive care unit

^a^Calculated for survival rate

Secondary efficacy outcomes were inflammatory biomarkers from BALF and blood samples. We found patients in the lobeline group had a greater decrease in procalcitonin (PCT) and C-reative protein (CRP) expressions (Table S3). Both the lobeline and control groups had comparable baseline levels of inflammatory and anti-inflammatory factors, except for serum TNF-α (Fig. 6b, Supplementary Fig. 8b). At 1 day after treatment initiation, we observed that BALF IL-1β, TNF-α, and serum IL-6 were obviously decreased, and IL-10 in BALF was significantly increased in lobeline group. A noticeable and statistically significant reduction in all inflammatory factors was observed in the lobeline group (Fig. 6b, Supplementary Fig. 8b). SpO2, which indicates the oxygen concentration in the blood, is an important physiological parameter of respiratory circulation; this value was significantly increased in ARDS patients within the first 24 hours of lobeline treatment (Supplementary Fig. 8c). Additionally, patients from both groups had pneumonia with bilateral radiographic opacities on chest radiography, and lobeline treatment might reduce inflammation foci (Supplementary Fig. 8d).

Taken together, intravenous infusion of lobeline was safe and could relieve lung inflammation within 24 hours in participants. To test the efficacy of lobeline for ARDS, larger trials will be needed.

## Discussion

In the present study, we demonstrated that the CAP is critical for MSC-mediated regulation of severe inflammatory responses, as demonstrated in a mouse model of ARDS. Inhibition of the CAP, either by blockade of vagus nerve, pharmacological inhibitors or genetic knockout, markedly decreased the therapeutic effects of MSCs. This regulation relied on MSC-derived PGE2, which greatly contributed to the expression of ACh-related synthases (Supplementary Fig. 9), indicating that activation of CAP could be a new approach for treating ARDS.

Most studies have attributed the therapeutic effects of MSCs to their secretion of soluble factors, including TGF-β,^36^ IDO,^36^ TSG-6,^36^ that exert modulatory effects on immune cells as well as to their reparative effects or antimicrobial effects.^13,37^ However, the studies have not yet fully explained how MSCs effectively reduce excessive inflammation and protect the lung from inflammatory cytokine-induced injury. The CAP modulates inflammatory responses, which is the potential therapeutic targets for inflammatory disorders. Previous study demonstrated that sciatic nerve activation with electroacupuncture efficiently inhibited systemic inflammation and saved mice from polymicrobial peritonitis.^38^ Lim HD et al. and Jin H et al. also observed that vagal nerve stimulation may regulate inflammatory responses.^39,40^ Moreover, the CAP modulated the inflammatory response via the vagus nerve by inhibiting inflammatory cytokine release via α7nAChR.^26^ Extracellular ACh targets mAChRs or nAChRs to play different physiological roles.^28^ Cholinergic system related immune cells, mononuclear leukocytes, and dendritic cells express several nAChR subunits (α2-α7, α9-α10) and five M1–M5 mAChR subtypes.^32^ In the present study, pulmonary inflammation was reduced 6 hours after MSC infusion and lung protection was partially reversed by α7nAChR antagonists. Thus, for the first time, this research has revealed that MSCs control inflammation by stimulating the CAP.

Several studies have shown that PGE2 was a vital regulator for the function of vagus nerve system,^41^ and mPGES-1 knockout mice showed the deficiency of ACh release.^42^ Here, we show that PGE2 secretion from MSCs play the important role for ACh release, and when PGES-2/PTGES are inhibited, MSCs exhibit decreased abilities to promote ACh production and inhibit inflammatory cytokine expression, highlighting its role in CAP activation. Moreover, Suenaga A reported that intracellular cAMP levels were increased via the PGE2 receptors EP2 and EP4 and could increase the mRNA expression and activity of ChAT.^43^ Therefore, previous reports and the present results together suggest that there might be a process promoted by MSC-derived PGE2 that facilitates CAP signaling in the lung.

The study supports the CAP pathway has the potential to become an innovative treatment strategy for acute lung injury and other inflammatory lung diseases. First, in lung injury mouse models in which the CAP was inhibited, MSCs distinctly decreased the anti-inflammatory effects, suggesting that the CAP mediates the effectiveness of MSCs in the treatment of ARDS. Second, we found that CAP-related nAChR and α7nAChR agonist treatments exert lung-protective effects and significantly improve inflammation in lung injury animal models, suggesting that these agents could be potential drug candidates for ARDS treatment. Therefore, we sought to identify cholinergic signaling-related clinical drugs; we identified the nicotinic acetylcholine receptor agonist lobeline, which found in Indian tobacco, is the predominant alkaloid in a family of structurally related compounds, and has shown potential as a respiratory stimulant in the treatment of asthma and respiratory illness.^34,44^ Lobeline acts through multiple mechanisms that are not yet well understood, and the drug has additional properties beyond its respiratory stimulant effects through stimulating chemoreceptors in the carotid body and aortic body. Kun-Cheng Li utilized lobeline to improve acute lung injury and reported that it could reduce the production of IL-6, NO via inhibiting the oxidative stress and NF-κB signaling pathway.^34^ Our results further demonstrated that intravenous administration of lobeline was well-tolerated in moderate to severe ARDS patients and could attenuate lung inflammation rapidly, suggesting lobeline may not only stimulates the respiratory center to accelerate respiration, but also activates the α7nAChR pathway to reduce inflammation, and play the role of killing two birds with one stone for treating ARDS.

In conclusion, we show that MSC therapy can suppress the excessive inflammatory response in ARDS, and this effect occurs through the CAP and the nAChR-dependent suppression of proinflammatory cytokine production. The modulation of ChAT expression are associated with the production of PGE2 by MSCs. Targeting neuroimmune interactions may be potential therapeutic targets for acute lung injury. Our findings provide novel insights for elucidating the mechanisms by which MSCs exert therapeutic effects on ARDS and suggest a potential treatment strategy.

## Materials and methods

### The ethics approval statements

All the animal experiments were approved by the Committee on the Ethics of Animal Experiments of Zhongshan School of Medicine, Sun Yat-sen University (No. 2018-202).

The observational study ([2019]02-531-01) was approved by the Research Ethics Board of the Third Affiliated Hospital of Sun-Yet Sun University. Informed consent was all obtained.

### Animals

8-10 weeks, male adult C57BL/6 mice used in this study were purchased from Guangdong Medical Laboratory Animal Center. *ChAT-IRES-Cre* mice (B6;129S6-Chattm2(cre)Lowl/J, JAX, Stock No: 006410), *Loxp-dTomato* mice (B6;129S6-Gt(ROSA)26Sortm9 (CAG-tdTomato)Hze/J, JAX, Stock No: 007905), and α7nAChR^−/−^ mice (B6.129S7-Chrna7tm1Bay/J, JAX, Stock No: 003232) were also used in the experiments. The mice were raised in individually ventilated cages under specific pathogen free (SPF) conditions.

### Experimental LPS-induced lung injury model and bacterial pneumonia model

Mice aged from 8 to 10 weeks were anesthetized by intraperitoneal administration of 8.1 µg xylazine and 90 µg ketamine per gram, and then, the mice were subjected to nasal inhalation of 5 mg/kg LPS (L2637, Sigma–Aldrich) from *Escherichia coli* (055:B5) in 50 μl saline, the sham control used an equal volume of saline. For bacterial pneumonia model, mice were exposed to nasal in halation of 10^6^ cfu *S. aureus* (BeNa Culture, BNCC353781), or 10^6^ cfu *E. coli* (BeNa Culture, BNCC125787) in 50 μl saline, the sham control used an equal volume of saline.^45,46^

### Isolation, characterization, and transplantation of MSCs

Derivation of MSCs was performed following previously described protocols.^47^ In brief, heparin-treated bone marrow of healthy donors with informed consent were isolated with Ficoll-Paque (1.077 g/mL, Amersham Biosciences) and cultured at 1 × 10^5^ cells/cm^2^; these cells were considered to be in passage 0. The cells were continuously passaged using 0.25% trypsin-EDTA when the cultures reached 80–90% confluence. For MSC administration, 1 × 10^6^ cells (passages 4-8) were suspended in 0.1 ml PBS and transplanted into mice via tail vein injection 6 hours after the mice were subjected to nasal inhalation of LPS.

### Anesthesia and resection of the vagus nerve

In preparation for surgery, the neck area was cleaned with alcohol and betadine alternately after the mice were anesthetized. An incision of approximately 5 mm was made on the left side of the throat, 2–3 mm from the clavicle. In order to visualize the carotid jugular bundle, the fatty tissue and fascia were gently separated from salivary glands. A small incision was made to expose the vagus nerve between the carotid artery and the jugular vein. For anesthetization, ~39.1 mg/kg ropivacaine (AstraZeneca) was injected into the vagus nerve.^48^ For resection, the vagus nerve was firmly grasped and pulled toward the head of the mouse until the vagus broke.

### Immunohistochemical analysis

Tissues were collected and fixed for 12 hours with 4% phosphate-buffered paraformaldehyde (PFA). 15-μm paraffin tissue slices were used for H&E staining. Following incubation for 60 minutes with anti-ChAT or anti-VAChT antibodies at 1:250 dilution, sections were treated with HRP-conjugated secondary antibodies for 30 minutes. Immunolabeled Ag-Ab complexes were visualized following the manufacturer’s instructions (Cell Signaling Tech.). Hematoxylin was used to counterstain with the sections before analysis.

### Vascular permeability

The amount of Evans blue dye accumulated in tissues was measured to determine the lung vascular permeability. A tail vein (i.v.) administration of Evans blue (25 mg/kg, Sigma-Aldrich) was administered 2 hours prior to lungs being harvested. Evans blue was extracted by incubating the lungs with 1 ml of formamide for 18 hours, at 60 °C, followed by perfusion in 5 ml PBS, homogenization in 1 ml PBS, and then washing twice. Centrifugation was used to separate the supernatant for 30 minutes at 5000 *g*. By using dual wavelength spectrophotometry at 740 nm and 620 nm, the amount of Evans blue was measured, and the concentration was calculated with the following equation: E620 (Evans blue) = E620 - (1.426×E740 + 0.03).^49^

### Lung injury score and BALF cell collection

The lung injury score was generated using five independent variables, including (A) neutrophils in the alveolar space, (B) neutrophils in the interstitial space, (C) hyaline membrane formation, (D) intra-alveolar proteinaceous material, and (E) alveolar septal thickening, which were weighted according to the relevance attributed to each characteristic, and then normalized to the number of fields evaluated. The relevant degree of lung injury score is a continuous value between zero and one (inclusive).^25^ The score was calculated as with the following formula: = [(20 × A) + (14 × B) + (7 × C) + (7 × D) + (2 × E)]/(number of 100 × fields). At least two pathologists who were blinded to the treatment conducted the histological assessments.

A cannula was inserted into each mouse’s trachea and 1.5 ml saline were infused into and out of the respiratory tract for three times to collect BALF and BALF cells. The cells in the BALF were stained with anti-CD11b, anti-CDF4/80, and anti-Ly-6G (1:50, eBioscience) antibodies for 30 min. Macrophages (CD11b + CDF4/80 cells) and neutrophils (CD11b + Ly-6G + cells) were sorted or analyzed by immunofluorescence staining and flow cytometry (MoFlo Astrios EQ/CytoFLEX).

### Enzyme-linked immunosorbent assay (ELISA)

Anti-mouse IL-1β, CXCL15, TNF-α, IL-6 ELISA kits (Invitrogen) were used to assess inflammatory factors concentrations in the mice with LPS-induced lung injury. Anti-human IL-1β, IL-6, TNF-α, and IL-8 kits (Invitrogen) were performed to assess the levels of inflammation in the BALF from ARDS patients.

### Bulk RNA-seq

TRI Reagent (#TR118, Molecular Research Center, Inc.) was used for the total RNA extraction according to the manufacturer’s protocol. RNA samples with an RNA integrity number >8 were used to prepare libraries, and paired-end sequencing was performed by CapitalBio Technology (China) following a standard protocol. Illumina sequencing raw data were demultiplexed and filtered using bcl2fastq2 and fastp. The data were then mapped to the Mouse Genome mm10 or Human Genome hg38 reference sequences, and the gene expression values were estimated using CLC Genomics Workbench 11.0 (Qiagen). The reads comprising a mixture of human and mouse sequences were disambiguated with Disambiguate (https://github.com/AstraZeneca-NGS /disambiguate). For some datasets, lists of neurotransmitter synthesis/transport, cytokine activity, and neuromodulator/neuromodulator biosynthetic process-related genes were acquired from the Ingenuity Pathway Analysis (IPA), Gene Ontology (GO), and NeuronDB databases, respectively. Selected genes expressed in at least one group were used to generate heatmaps, which were produced using HemI 1.0.

### Immunofluorescence analysis

After 2 days of lung injury, the mice were euthanized and perfused with ice-cold saline followed by 4% PFA. Lung samples were collected, fixed overnight in 4% PFA, and cut into 15-μm frozen cryosections. The pulmonary sections were permeabilized with 0.15% Triton X-100 for 15 min, blocked with 5% normal goat serum in PBS for 1 hour, incubated overnight at 4 °C with anti-VAChT antibodies. Incubation with secondary antibodies was done for 30 min at room temperature before staining the nuclei with DAPI (Fluka) for 10 min. Immunofluorescent images were acquired using an LSM 880 confocal microscope (Zeiss).

### Quantitative real-time polymerase chain reaction (qRT–PCR)

According to the manufacturer’s protocols, total RNA was extracted from lung tissues of each group with an RNeasy mini kit (Qiagen). Using a RevertAid First Strand cDNA Synthesis Kit (Thermo Fisher Scientific), cDNA was generated with 1 μg RNA sample. qRT–PCR was performed in duplicate with SYBR Green qRT–PCR Super Mix (Roche), and the reactions were performed with a Light Cycler 480 Detection System (Roche). The primers used for the qRT–PCR analysis are listed in Supplementary Table 4.

### Western blotting

Lung tissue homogenates were collected from each group. CD11b + F4/80+ cells were isolated from BALF using FACS and lysed with 1X RIPA buffer for protein extraction. A BCA protein assay kit was used to determine the protein concentrations (Thermo Fisher Scientific). Protein samples were separated with SDS–PAGE gels and then transferred to polyvinylidene fluoride (PVDF) membranes with 0.45-μm or 0.22-μm pores (Millipore). PVDF membranes were blocked with 5% BSA, and incubated with the appropriate antibodies. The antibodies used for Western blotting analysis are listed in Supplementary Tables 5 and 6.

### Liquid chromatography–tandem mass spectrometry (LC-MS/MS)

After pretreatment with acetonitrile, the target analyte ACh in BALF, serum and cell culture supernatant were identified and quantified by ULTRA-high pressure LC-MS/MS System (SCIEX Triple Quad 5500+). Briefly, the analytes were separated on an Agilent Poroshell 120 HilIC-Z (2.1*100 mm, 2.7μm) liquid chromatographic column. The solvents were 5 mmol/L ammonium formate −0.1% formic acid water (solvent A) and 5 mmol/L ammonium formate +90% acetonitrile aqueous solution (solvent B). The gradient during the running time of 10 min was as follows: 10%A (initial), 10–10%A (0–0.5 min), 10–40%A (0.5–1.25 min), 40–40%A (1.25–1.7 min), 40–10%A (1.7–1.71 min), 10–10%A (1.71–10 min). All gradient steps are linear. Mass spectrometry conditions: electrospray ion source (ESI), positive ion MRM scanning mode, 400 ^o^C, 35 psi of curtain gas, 5500 V of ion spray voltage, 9 psi of collision gas, 50 psi of auxiliary gas 1, 50 psi of auxiliary gas 2. ACh quantitative ion pair 146.1/87.1, cluster removal voltage 80 V, collision energy 21.88 V; Qualitative ion pair 146.1/60.1, cluster removal voltage 80 V, impact energy 15.18 V. BALF, serum and cell culture supernatant were stored at −80 ^o^C until analysis.

### Flow cytometry

Experimental mice were euthanized and BALF cells were collected. For further immunostaining and FACS analysis, single-cell suspensions were collected from BALF. BALF cell suspensions were stained with antibodies against F4/80, CD11b, Ly6G, TNF-α, and IL-1β. Macrophage suspensions were stained with primary antibody against α7nAChR for 1 hour, and then the secondary antibody was incubated for 30 min. Macrophages with only a second antibody staining were used as controls.

For BALF cells obtained from patients, suspensions were stained with antibodies against CD11b, CD14, TNF-α, IL-8, and IL-1β. CytoFLEX flow cytometer was used to perform flow cytometry experiment, and the results were assessed with the CytExpert (Beckman) and FlowJo X 10.0.7r2 software packages (BD). The antibodies utilized for flow cytometry are listed in Supplementary Tables 7 and 8.

### Vagus neurons dissociation

For each individual experiment, a minimum of 20 mice were anesthetized and performed transcardial perfusion with cold standard, bubbled with 95% O_2_–5% CO_2,_ artificial cerebrospinal fluid (ACSF) solution.^50^ Neurons from vagus ganglia could be collected and placed in ice-cold Ca^2+^ and Mg^2+^-free Hank’s Balanced Salt Solution supplemented with penicillin–streptomycin. The culture medium consisted of 21.5 mls of Ham’s F-12 Nutrient Mix (GIBCO); 21.5 mls of Neurobasal A (GIBCO); 5mls Glutamine; 1 ml of B-27 Supplement (GIBCO); 1 ml of Penicillin-Streptomycin solution (Thermo). The ratio of MSCs to neurons was 10:1. Recombinant human IL-1β (100 ng/ml, R&D systems) and mouse IL-1β (100 ng/ml, R&D systems) were used to stimulate the PGE2 production of MSCs. Indomethacin (10 nM, MedChem Express) and MF63 (10 nM, MedChem Express) were used to inhibit PGE2 synthetase, PTGS2 and PTGES, respectively.

### Transfection with siRNA

To knockdown PTGS2 and PTGES expression, using Lipofectamine^®^ RNAiMAX Reagent (13778-150; Thermo Fisher Scientific), human MSCs were transfected with PTGS2 siRNA (RiboBio) and PTGES siRNA (RiboBio), respectively. After transfection, MSCs were cultured for 48 hours, and qRT–PCR was performed to determine the knockdown efficiency. The siRNA sequences were as follows:

PTGS2 sequence 1: 5′-GGGTAATGTTATATGTTCTdTdT-3′.

PTGS2 sequence 2: 5′-GGAACGTTGTGAATAACATdTdT-3′.

PTGES sequence 1: 5′-GGAACGACATGGAGACCATdTdT-3′.

PTGES sequence 2: 5′-GGGCTTCGTCTACTCCTTTdTdT-3′.

### mAChR/nAChR agonists and antagonists intervention

The peripheral mAChR agonist Arecoline hydrobromide, mAChR antagonist Methylbenactyzium bromide, and nAChR agonist Monepantel, nAChR antagonist Vinblastine were obtained from MedChem Express (New Jersey, USA). The α7nAChR agonists AR-R17779 hydrochloride and GTS-21, antagonist Methyllycaconitine citrate were also purchased from MedChem Express (New Jersey, USA. The reagents were administered 4 hours after LPS instillation once (5 mg/kg dose of the reagents). Stored at −20 ^o^C, all the reagents were diluted in saline to the final concentration and were injected intraperitoneally.

### BALF samples from ARDS patients

Patients with ARDS (see Supplementary Table 1) were strictly recruited according to the Berlin definition^35^ at a surgical ICU from 2019 to 2022. BALF samples were collected before and after lobeline treatment. All the procedures were performed in accordance with the Hippocratic oath. This study strictly conformed to the ethics guidelines of the 1975 Declaration of Helsinki and was reviewed. This clinical trial has been registered in the Chinese Clinical Trial Registry (ChiCTR2100047403).

### Statistical analysis

All the results in this study are presented as the mean ± SD of at least three independent experiments. The sample size of each experiment is indicated in the corresponding figure legend. Statistical analyses were performed with SPSS version 22 software using one-way ANOVA when several groups were being compared or unpaired Student’s *t* test (two-tailed) when two groups were being compared. Log-rank test was used to compare survival curves. It is considered to be statistically significant when *P* values are less than 0.05.

## Supplementary information

supplemental material

## Data Availability

The datasets generated and/or analyzed during the current study are available from the corresponding author upon reasonable request.
