# Supplementary material for: Mesenchymal stromal cells alleviate acute respiratory distress syndrome through the cholinergic anti-inflammatory pathway
Source: Signal Transduct Target Ther. 2022 Sep 5;7:307. doi: 10.1038/s41392-022-01124-6 (PMC9441842; doi:10.1038/s41392-022-01124-6)

Supplementary Materials for

Mesenchymal Stromal Cells Alleviate Acute Respiratory Distress Syndrome through the Cholinergic Anti-Inflammatory Pathway

Xiaoran Zhang, Xuxia Wei, Yiwen Deng, Xiaofeng Yuan, Jiahao Shi, Weijun Huang, Jing Huang, Xiaoyong Chen, Shuwei Zheng, Jieying Chen, Keyu Chen, Ruiming Xu, Hongmiao Wang, Weiqiang Li, Shiyue Li, Huimin Yi*, Andy Peng Xiang*

Correspondence to: xiangp@mail.sysu.edu.cn&ylhmin@hotmail.com

**This PDF file includes:**

Figures. S1 to S9

Tables S1 to S8


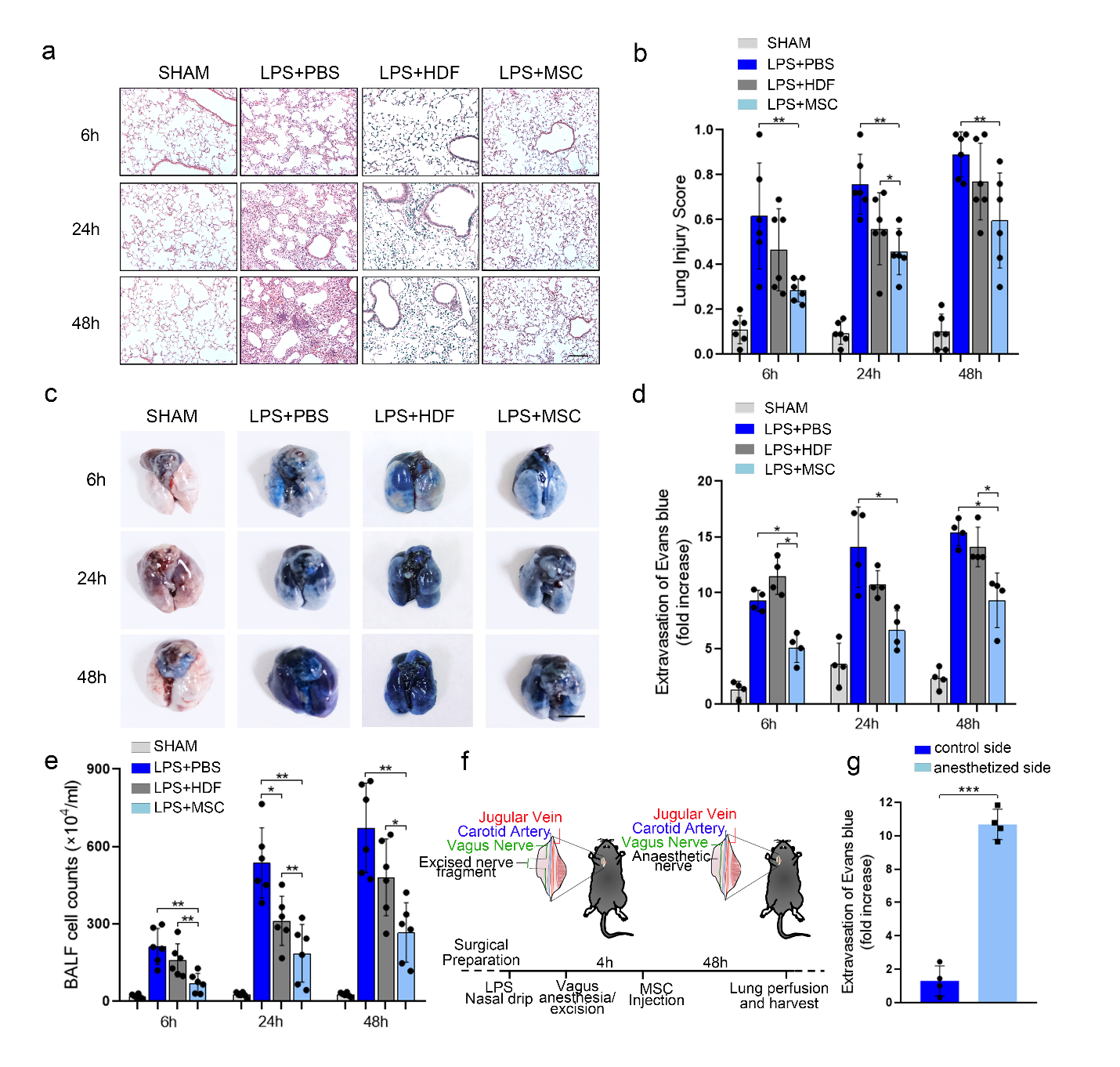


Figure. S1. MSC treatment improves LPS induced lung injury.

(a) Representative H&E-stained lung samples from mice of each group. 6 hours, 24 hours, 48 hours post-injection, lung tissues were collected, paraffin-embedded, sectioned, and stained with H&E. Photos were taken of at least six sections per tissue. Scale bars, 100 μm.

(b) Lung injury score was calculated according to H&E-staining.

(c) Photographs depicting lung vascular permeability was assessed by Evans blue accumulation in the lung tissue. Evans blue dye was injected via the tail vein at 6 hours, 24 hours, 48 hours after lung injury. The levels of Evans blue dye extravasation into the lung tissue was shown in picture.

(d) Lung tissues from four groups were collected, and the levels of Evans blue dye were assessed by measuring absorbance at 620 nm and 740nm.

(e) The infiltration of leucocytes in BALF were compared with each group.

(f) Schematic depiction of the right vagus anesthetization or right vagotomy surgery in LPS-induced lung injury model mice.

(g) Lung tissues from Ropivacaine treatment side and control side were collected, and the levels of Evans blue dye were assessed by measuring absorbance at 620 nm and 740nm.

*p < 0.05, **p < 0.01, ***p < 0.001.


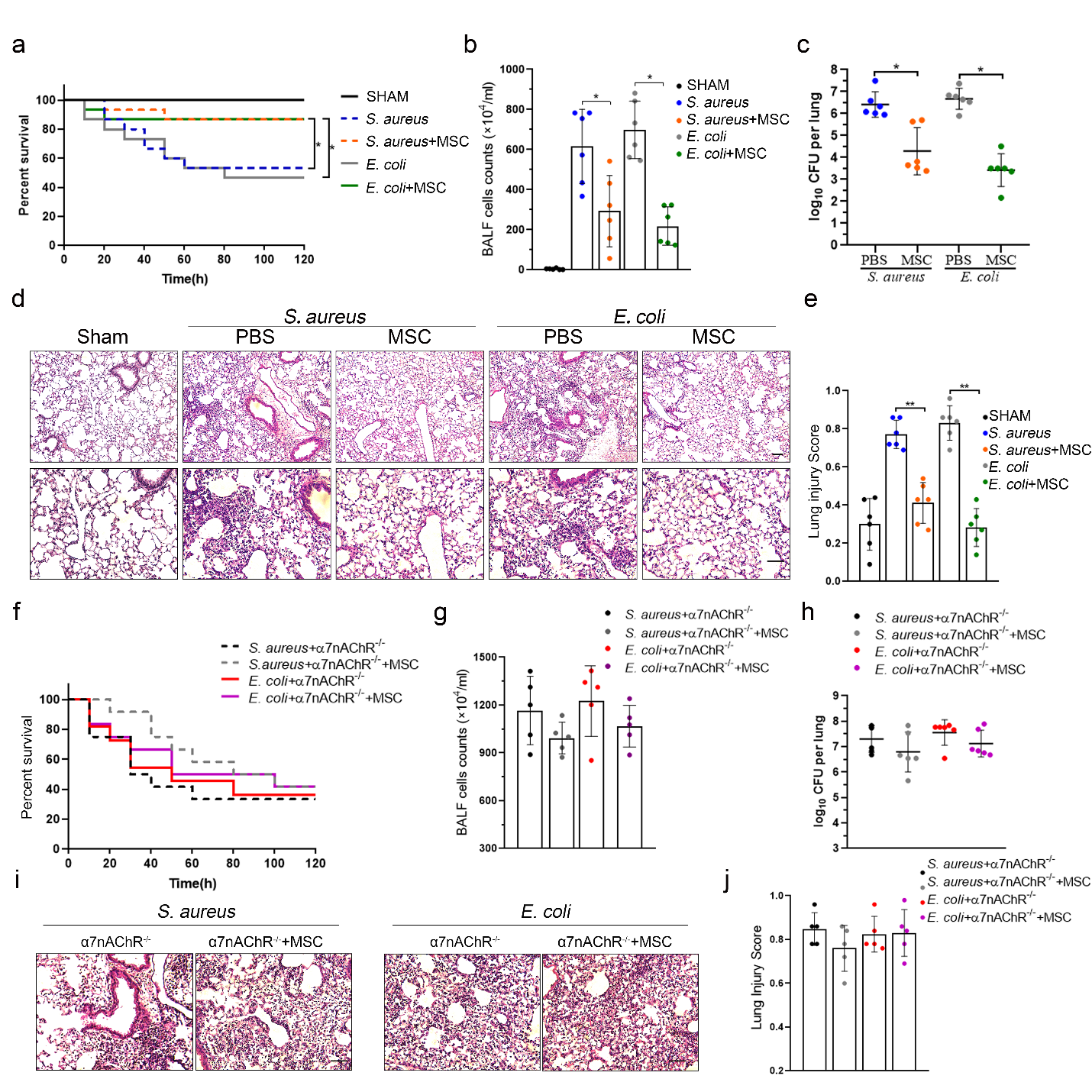


**Figure. S2. MSC treatment improves lung injury via the CAP in bacterial-caused pneumonia.**

(a) 2 hours after *S. aureus* or *E. coli* stimulation, mice were treated with either PBS or MSCs, and mice were kept alive for observation of their viability and behavior for 120 hours, and showed survival curves for mice from each group.

(b) Quantification of total cell number in the BALF from different groups; n=6 mice per group.

(c) Mice were scarified 12 hours after infection for the evaluation bacterial loads (CFU counts in whole lung homogenize); n=6 mice per group.

(d) Representative H&E-stained lung samples from mice of each group. Lung tissues were collected, paraffin-embedded, sectioned, and stained with H&E. Photos were taken of at least six sections per tissue sample. Scale bars, 100 μm.

(e) Quantitative analysis of lung injury score; n=6 mice per group.

(f) 2 hours after *S. aureus* or *E. coli* stimulation, α7AChR^-/-^ mice were treated with either PBS or MSCs, and mice were kept alive for observation of their viability and behavior for 120 hours, and showed survival curves for mice from each group.

(g) Quantification of total cell number in the BALF from different groups; n=5 mice per group.

(h) Mice were scarified 12 hours after infection for the evaluation bacterial loads (CFU counts in whole lung homogenize); n=6 mice per group.

(i) Representative H&E-stained lung samples from mice of each group. Lung tissues were collected, paraffin-embedded, sectioned, and stained with H&E. Photos were taken of at least six sections per tissue sample. Scale bars, 100 μm.

(j) Quantitative analysis of lung injury score; n=5 mice per group.


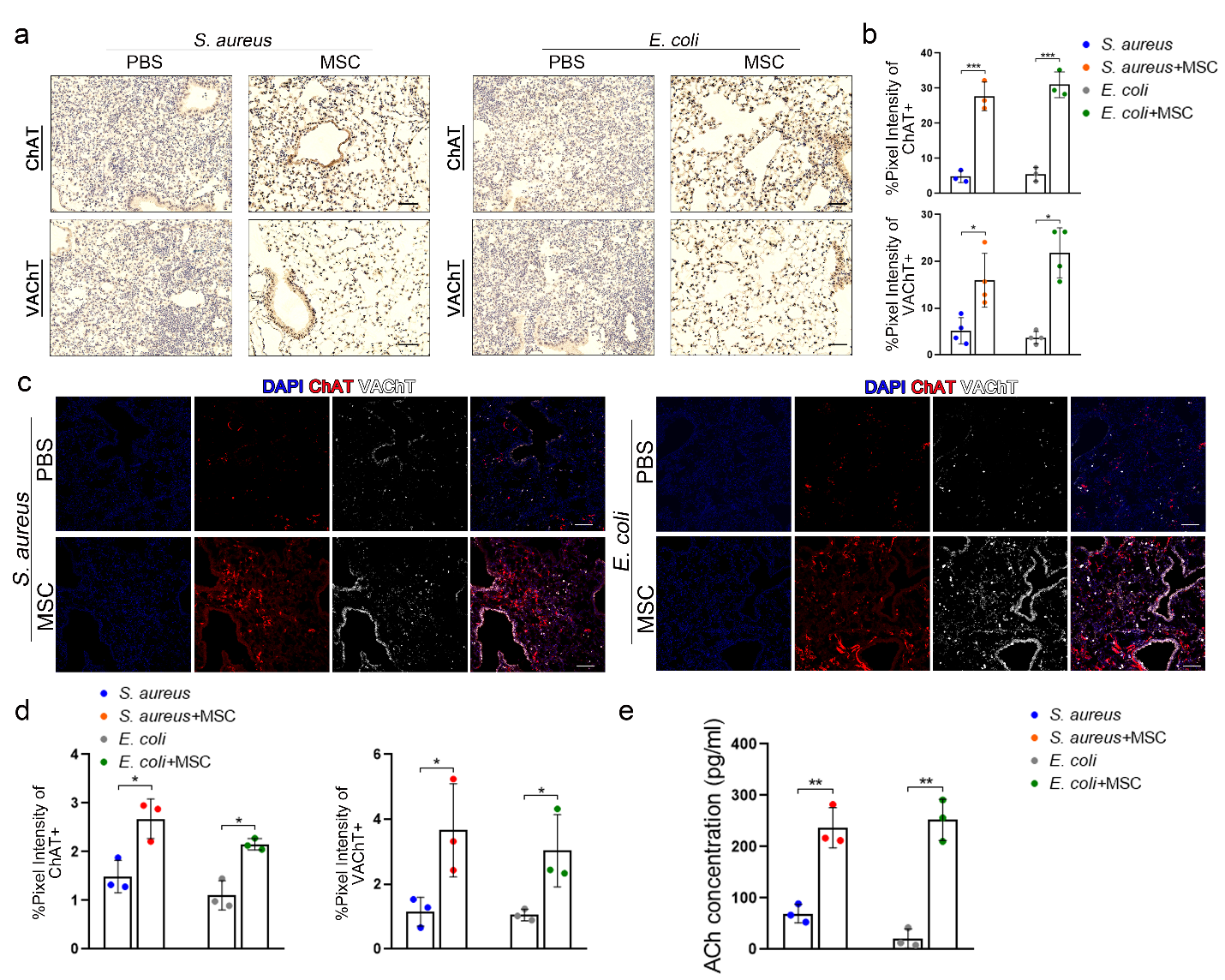


**Figure. S3. MSCs facilitate the upregulation of ChAT and VAChT expressions in bacterial-caused pneumonia.**

(a) Representative immunohistochemical staining of ChAT or VAChT pixel intensity; scale bars, 100 μm; n=3 mice per group.

(b) Representative immunohistochemical staining quantification of ChAT or VACHT pixel intensity.

(c) Representative confocal images of lung sections from different groups stained for MSCs (green) and ChAT (red) or VAChT (white). Nuclei were visualized by DAPI staining (blue). Scale bars, 100 μm.

(d) Quantification of the percentage of ChAT or VAChT pixel intensity; n=3 mice per group.

(e) The concentrations of ACh in BALF were measured by liquid chromatography–tandem mass spectrometry (LC-MS/MS); n=3 mice per group.


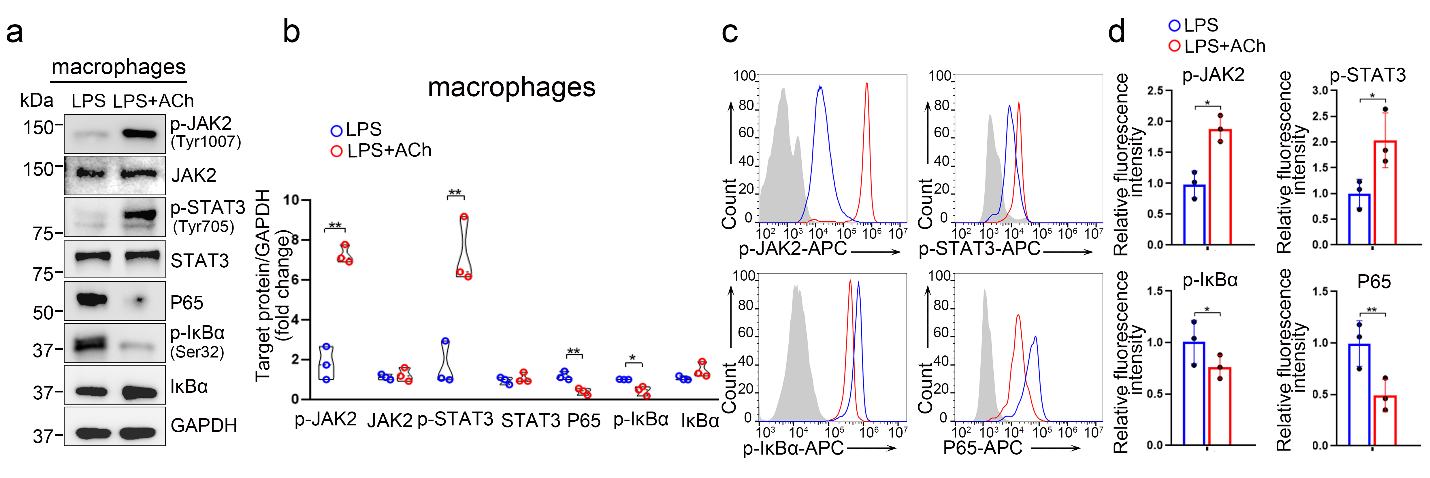


**Figure. S4. ACh regulating CAP signaling in relation to the JAK2/STAT3 and NF-kB pathways in macrophages.**

(a, b) Cell lysates of macrophages were analyzed by Western blotting of the listed phosphorylated proteins, with GAPDH detected as a loading control. A representative blot is shown (a). The comparison of the gray-scale values in (b) are shown. n=3 samples per group.

(c, d) Flow cytometric analysis of the listed phosphorylation levels of macrophages (c) and quantification (d) of the listed phosphorylation levels determined by mean fluorescence intensity (MFI) analysis. n=3 samples per group.

**
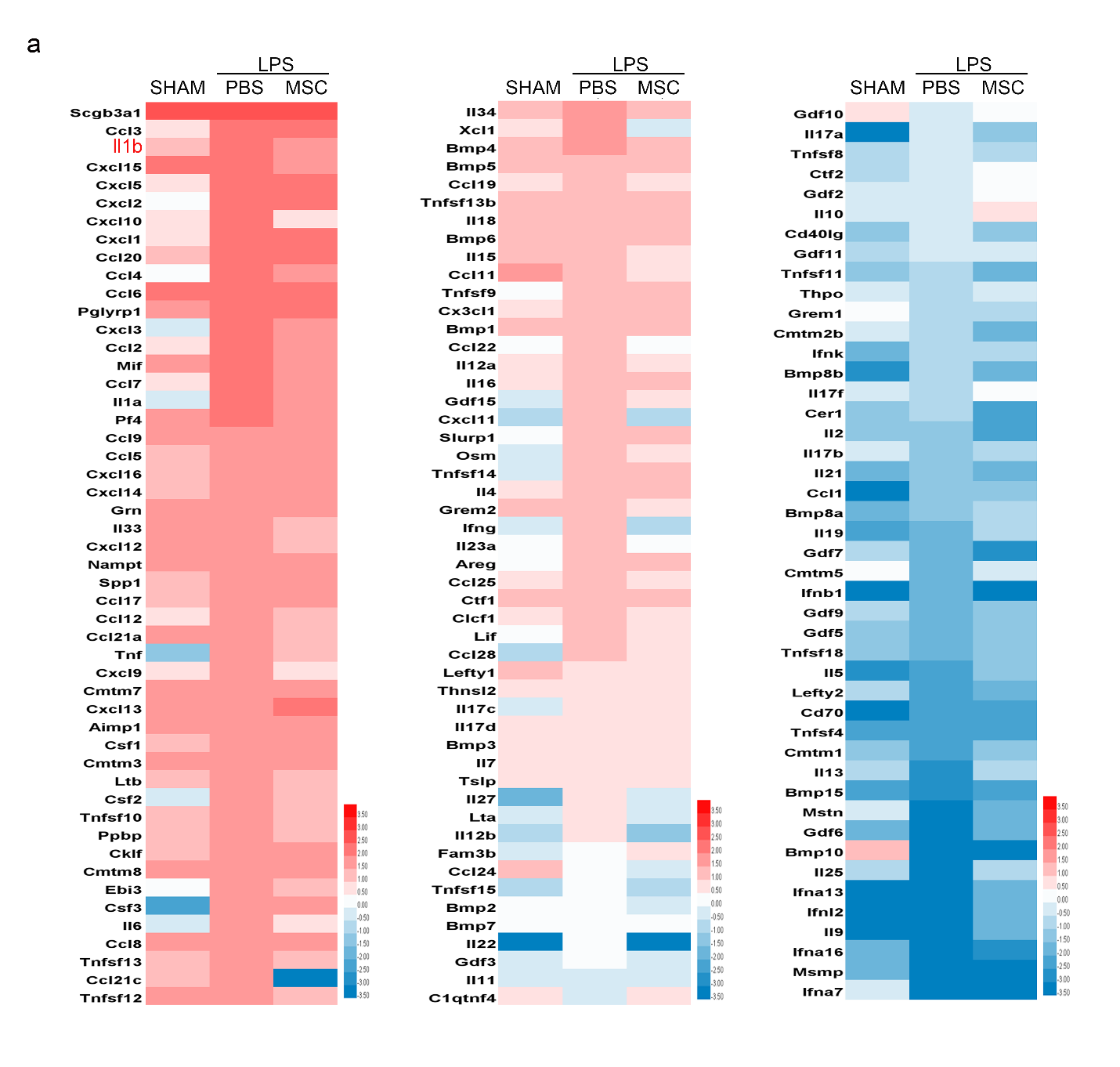
**

**Figure. S5. MSCs activate the CAP partially through COX-2/PGE2 pathway.**

(a) The genes of cytokine activity biosynthetic process related genes were ranked according to the expression changes in PBS treatment group. The expression values of cytokine activity biosynthetic process related genes were normalized with Z score before heatmap drawing.

**
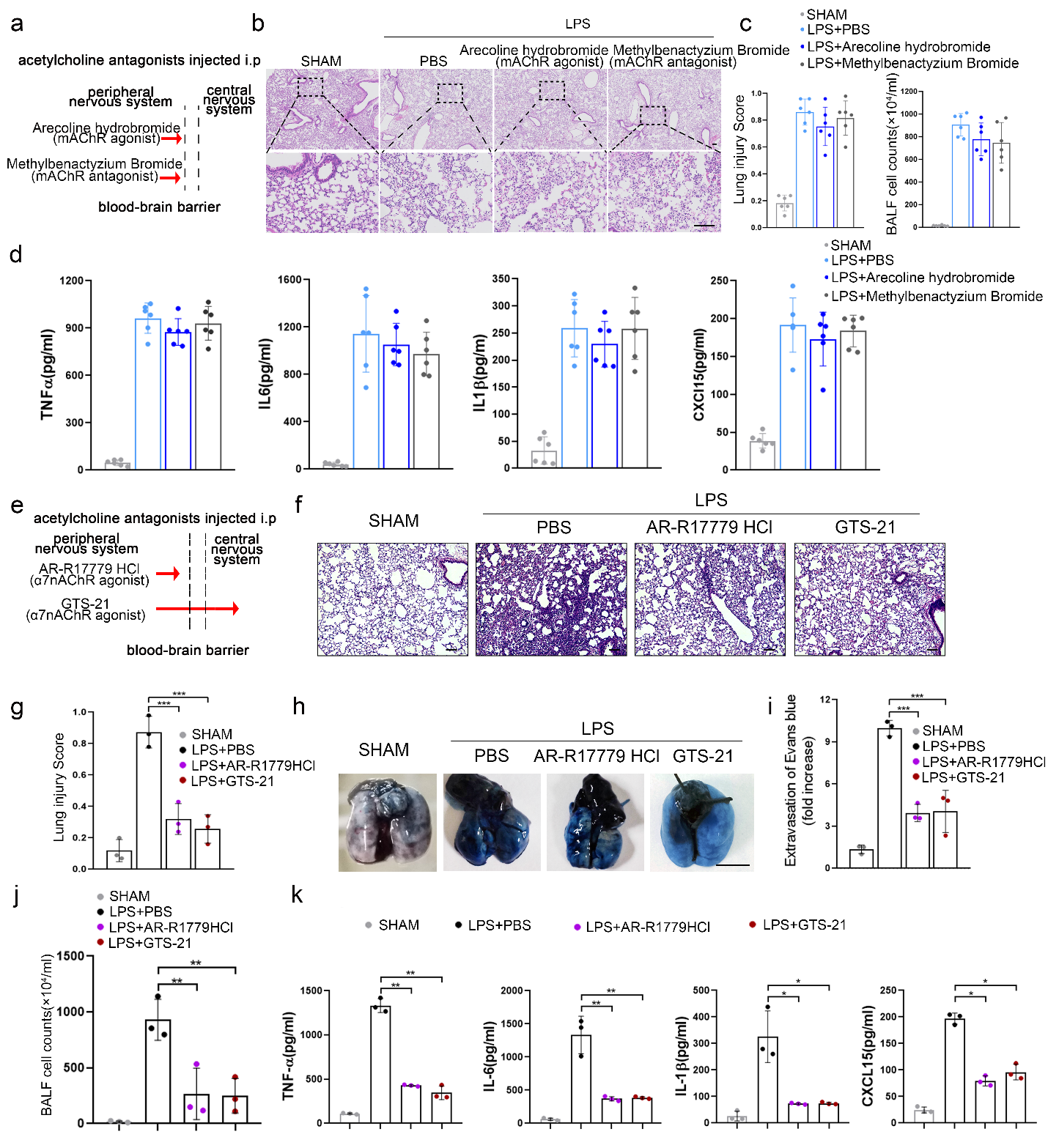
**

**Figure. S6. Effects of α7nAChR agonists in treating LPS-induced lung injury.**

(a) Peripheral mAChR agonist and antagonist, Arecoline hydrobromide and Methylbenactyzium Bromide were used to test the therapeutic effects in LPS-induced lung injury.

(b) Representative H&E-stained lung samples from mice of each group. Lung tissues were collected, paraffin-embedded, sectioned, and stained with H&E. Photos were taken of at least six sections per tissue. Scale bars, 100 μm.

(c) Lung injury score was calculated according to H&E-staining and the infiltration of leucocytes in BALF were compared with each group.

(d) The concentrations of TNF-α, IL-6, IL-1β, and CXCL15 in the BALF were measured by ELISA.

(e) Peripheral α7nAChR agonist AR-R17779 HCl and central α7nAChR agonist GTS-21 were used to test the therapeutic effects in LPS-induced lung injury.

(f) Representative H&E-stained lung samples from mice in each group. Lung tissues were collected, paraffin-embedded, sectioned, and stained with H&E. Photos were taken of at least six sections per tissue. Scale bars, 100 μm.

(g) The lung injury score was calculated based on H&E staining results, and the infiltration of leukocytes into BALF was compared among the groups; n=3 mice per group.

(h) Photographs depicting lung vascular permeability was assessed by Evans blue accumulation in the lung tissue. Evans blue dye was injected via the tail vein. The levels of Evans blue dye extravasation into the lung tissue was shown in picture.

(i) Lung tissues from four groups were collected, and the levels of Evans blue dye were assessed by measuring absorbance at 620 nm and 740nm.

(j) The infiltration of leucocytes in BALF were compared with each group.

(k) The concentrations of TNF-α, IL-6, IL-1β, and CXCL15 in BALF were measured by ELISA; n=3 mice per group.


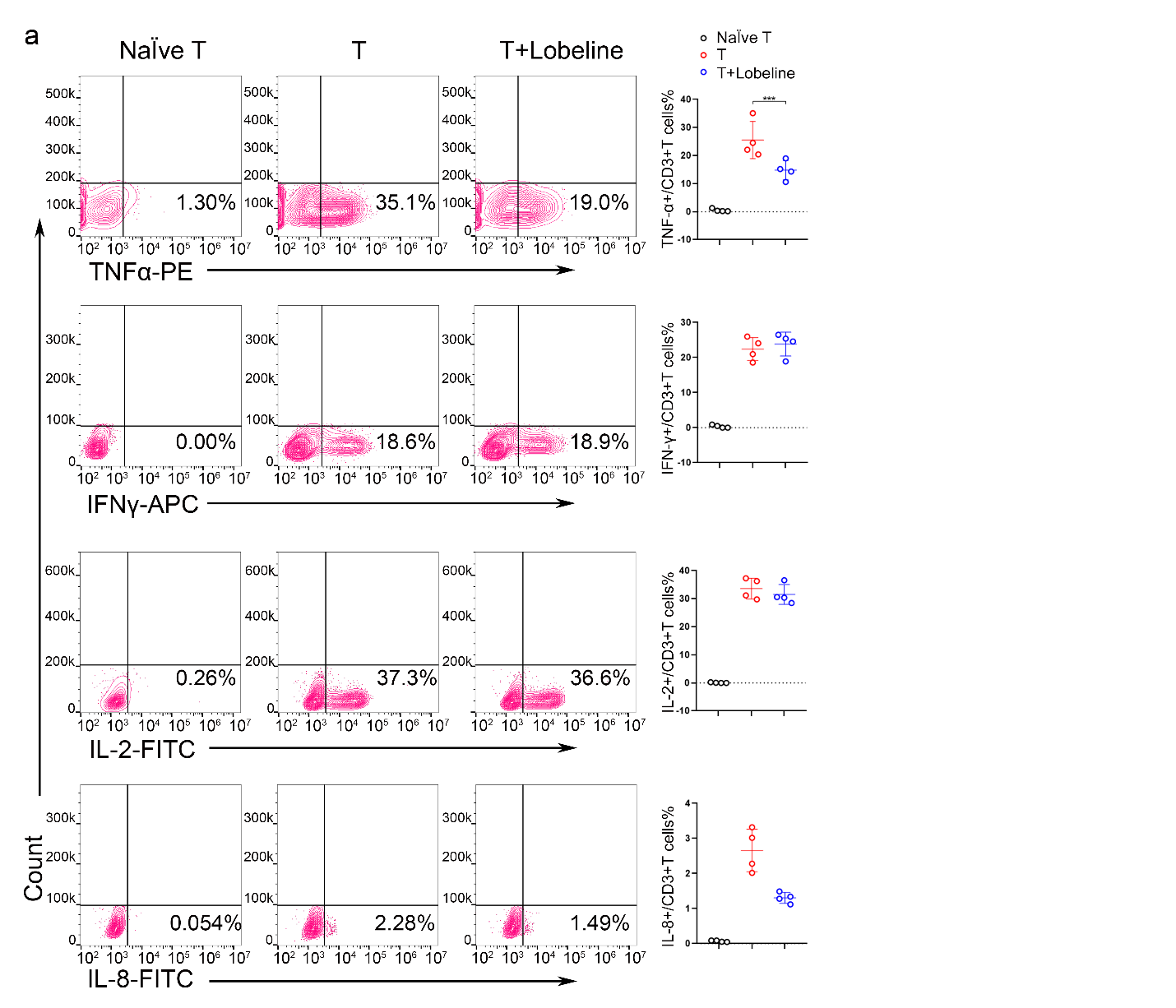


**Figure. S7. The potential anti-inflammatory effects of lobeline in treating lung injury.**

(a) The T cells in PBMCs (peripheral blood monouclear cells) were collected and cocultured with Lobeline, and the production of TNF-α, IFN-γ, IL-2, IL-8 were examined by flow cytometry.

**
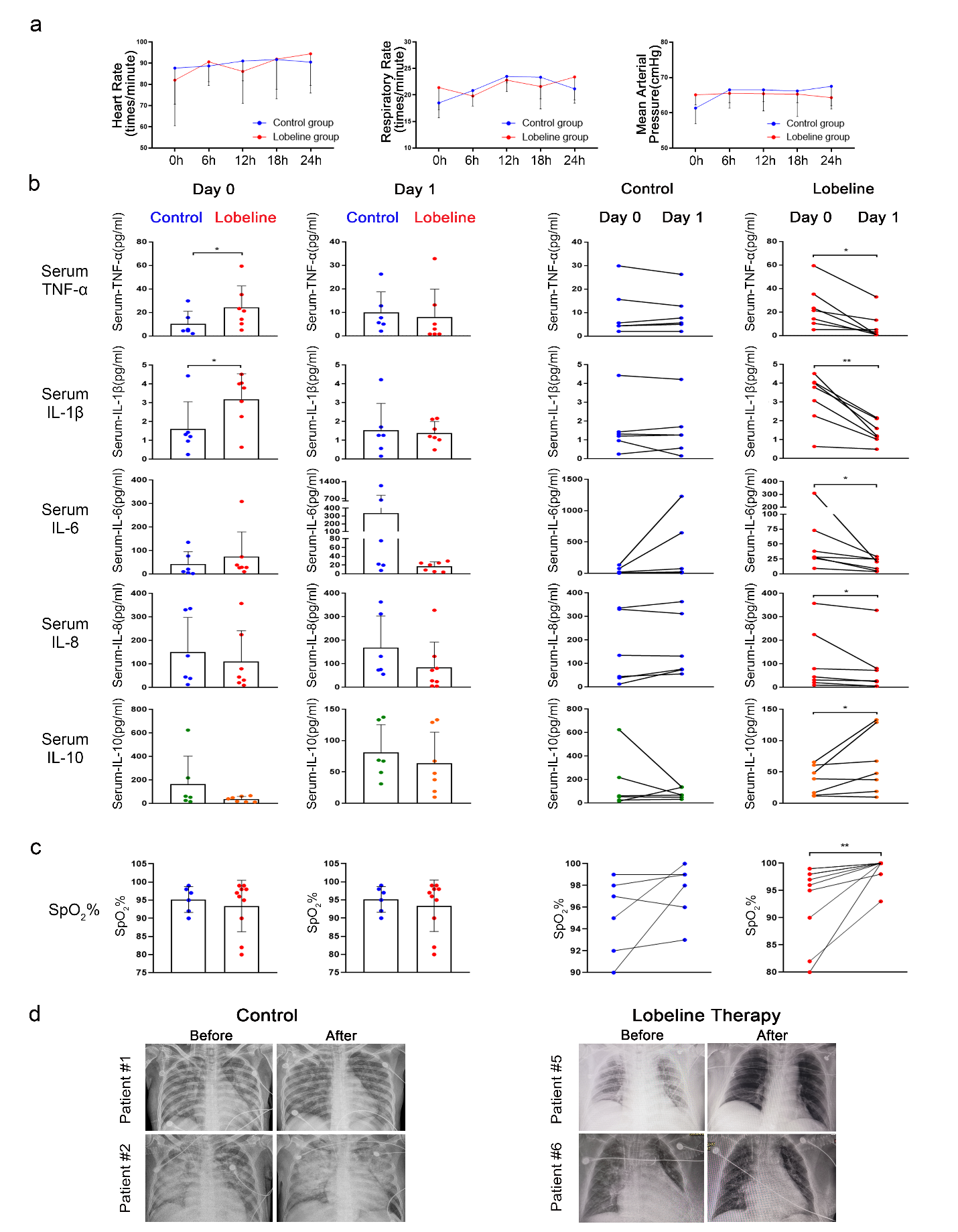
**

**Figure. S8. Lobeline improves the respiratory symptoms of ARDS patients.**

(a) Mean (SED) values of variation for heart rate (beats per minute), respiratory rate (times per minute), mean arterial pressure (cmHg) from control and lobeline group were recorded at 0, 6, 12, 18, 24 hours after treatment.

(b) Analysis of inflammatory cytokines in serum from two group patients. The concentrations of TNF-α, IL-8, IL-1β, IL-6, IFN-γ, and IL-10 in BALF were measured by ELISA. In the comparison between groups at day 0 and in the longitudinal analysis from day 0 to day 1, inflammatory cytokine concentrations showed marked and statistically significant decreases from day 0 to day 1 only in the lobeline treatment group.

(c) Mean (SED) values for arterial oxygen saturation, as measured at day 0 to day 1, use of a pulse oximeter (SpO2; %) from two group.

(d) Representative chest radiographs of patients with ARDS before and after routine or lobeline therapy, from control and lobeline group, respectively.


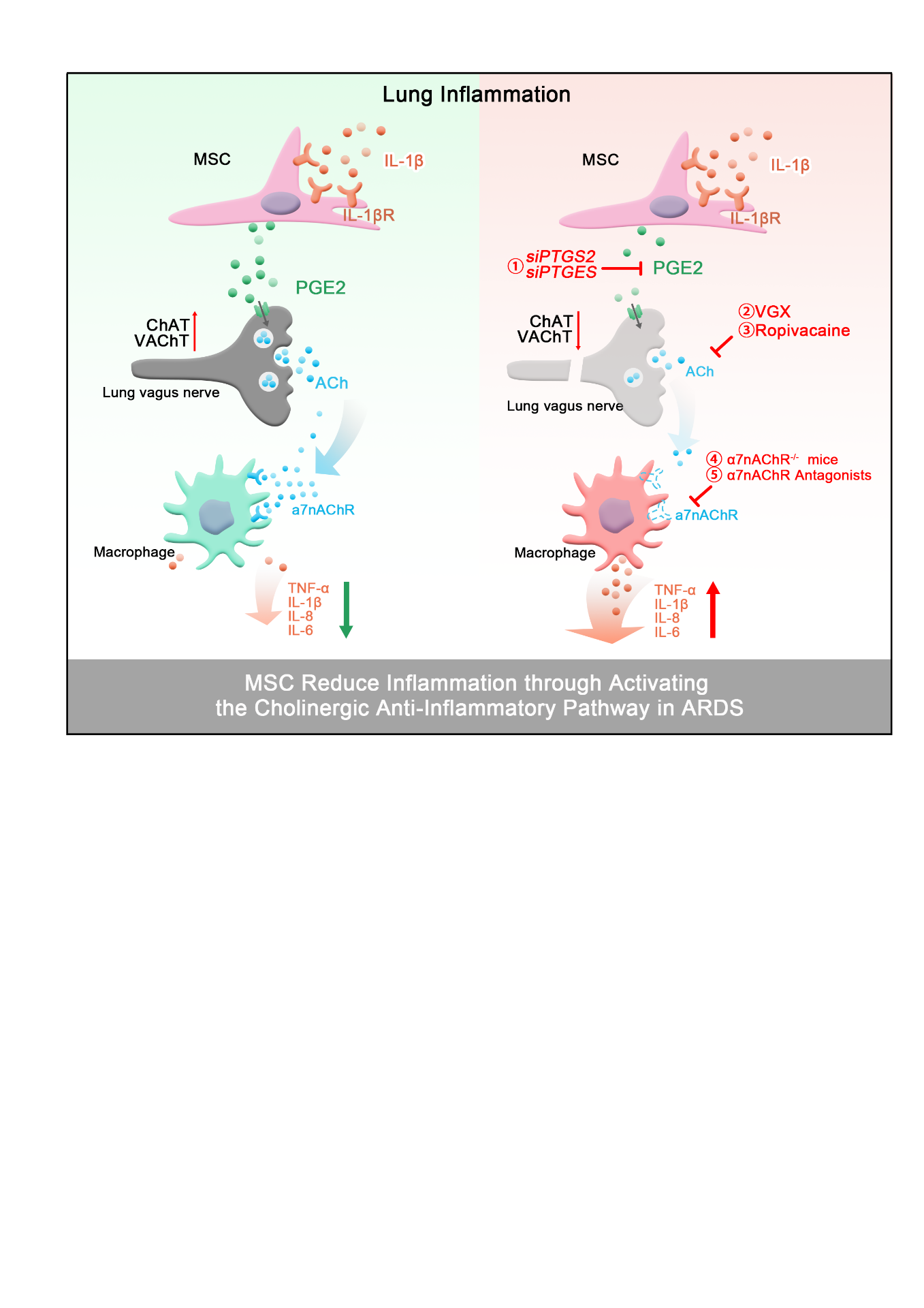


**Figure. S9. MSC treatment improves acute lung injury via the CAP.**

**Table S1**. **Enrollment rate of 16 patients with moderate/severe ARDS from the Third Affiliated Hospital of Sun Yat-sen University.**

| **Period** | **Number of randomized patients** |
| --- | --- |
| 2019 | 5 |
| 2020 | 0 |
| 2021 | 4 |
| 2022 | 7 |

**Table S2**. **Baseline characteristics at the time of ARDS diagnosis of 16 patientsᵞ.**

| **Characteristics** | **Control group**  **N=6** | **Lobeline group**  **N=10** |
| --- | --- | --- |
| Age, years | 59±12 | 60±13 |
| Male, no. (%) | 4(66.7) | 6(60.0) |
| SOFA score, mean±SD ¶ | 16.33±2.75 | 13.4±2.50 |
| PaO_2_/FiO_2_, mmHg | 132.5±33.41 | 143.3±29.50 |
| Tidal volume, mL/kg PBW | 6.53±0.41 | 6.84±0.46 |
| Respiratory rate, breaths/min | 19.17±1.57 | 20.9±5.79 |
| Positive end-expiratory pressure, cmH_2_O | 9.50±0.50 | 10.1±1.81 |
| Inspiratory plateau pressure, cmH_2_O | 26.17±0.69 | 28.4±2.91 |
| Days from ICU admission to ARDS diagnosis | 2.43±0.83 | 3.09±1.11 |
| Days from intubation to ARDS diagnosis | 1.16±0.35 | 1.14±0.44 |

ᵞ Plus-minus values are mean ± SD. There were no significant differences between the groups. PBW denotes predicted body weight.

¶Sequential Organ Failure Assessment (SOFA) score was measured in 6 organ systems (cardiovascular, respiratory, hematologic, hepatic, renal, and neurological system), with each organ scored from 0 to 4, resulting in an aggregated score ranging from 0 to 24, with higher scores indicating greater dysfunction.

|  | **Control group** | | | |  | **Lobeline group** | | | |
| --- | --- | --- | --- | --- | --- | --- | --- | --- | --- |
|  | 0 h | 24 h | Mean difference(95%CI) | P-value |  | 0 h | 24 h | Mean difference(95%CI) | p value |
| **PCT ng/ml** | 0.52±0.39  (n=6) | 1.6±2.43  (n=6) | -1.12  (-3.99 to 1.75) | 0.36 |  | 25.64±38.14  (n=10) | 11.82±21  (n=10) | -24.6  (-53.39 to 4.12) | 0.012* |
| **IL-6 pg/ml** | 172.15±155.78  (n=6) | 219.23±157.80  (n=6) | -47.07  (-124.89 to 30.735) | 0.181 |  | 157.31±138.76  (n=5) | 72.47±73.70  (n=5) | 84.84  (-48.77 to 218) | 0.15 |
| **CRP**  **mg/L** | 40.03±29.62  (n=6) | 47.38±45.82  (n=6) | -7.35  (-48.20 to 33.50) | 0.663 |  | 179.57±102.91  (n=10) | 104.83±103.83  (n=10) | 74.74  (2.57 to 146.91) | 0.044* |

**Table S3**. **Inflammatory factor biomarker changes.**

Data were present with mean±standard deviation, with t-test or nonparametric test for paired samples for analysis. Control and lobeline group inflammatory factor biomarker change differences between 0h and 24h were significant when *P*<0.05*.

**Table S4. Sequence of Specific Primers Used for qPCR Analysis:**

**Mouse:**

| **Genes** | **Forward Sequence** | **Reverse Sequence** |
| --- | --- | --- |
| GAPDH | TCAATGAAGGGGTCGTTGAT | CGTCCCGTAGACAAAATGGT |
| ChAT | CCGGTTGGTGGAGTCTTTTA | CAACGAGGATGAACGCCT |
| VAChT | CGGTTCATCAAGCAACACAT | CACTCACTTGGCTTTGAGCA |

**Human:**

| **Genes** | **Forward Sequence** | **Reverse Sequence** |
| --- | --- | --- |
| GAPDH | GAAGGTGAAGGTCGGAGTC | GAAGATGGTGATGGGATTTC |
| PTGS2 | CGGTGAAACTCTGGCTAGACAG | GCAAACCGTAGATGCTCAGGGA |
| PTGES | GAGGATGCCCTGAGACACGGA | CCAGAAAGGAGTAGACGAAGCC |

**Table S5. Primary Antibodies Used for Western Blot**

| **Antigen** | **Host** | **Source** | **Dilution** | **Identifier** |
| --- | --- | --- | --- | --- |
| ChAT | Rabbit | Invitrogen | 1:1000 | PA5-77838 |
| VAChT | Mouse | Invitrogen | 1:1000 | MA5-27662 |
| p-JAK2 | Rabbit | Cell Signaling Technology | 1:1000 | 4406T |
| JAK2 | Rabbit | Cell Signaling Technology | 1:1000 | 3230T |
| p-STAT3 | Rabbit | Cell Signaling Technology | 1:2000 | 9145T |
| STAT3 | Mouse | Cell Signaling Technology | 1:1000 | 9139T |
| P65 | Rabbit | Cell Signaling Technology | 1:1000 | 8242S |
| p-IκBα | Rabbit | Cell Signaling Technology | 1:1000 | 2859S |
| IκBα | Mouse | Cell Signaling Technology | 1:1000 | 4814S |
| GAPDH | Mouse | Cell Signaling Technology | 1:2000 | 97166S |

**Table S6. Secondary Reagents Used for Western Blot**

|  | **Source** | **Dilution** |
| --- | --- | --- |
| Anti-rabbit | Cell Signaling Technology | 1:2000 |
| Anti-mouse | Cell Signaling Technology | 1:2000 |

**Table S7. Primary Antibodies for Flow Cytometry**

| **Antigen** | **Source** | **Dilution** | **Clone** |
| --- | --- | --- | --- |
| α7nAChR | abcam | 1:500 | ab216485 |
| Anti- mouse CD11b (FITC) | Biolegend | 1:50 | 101206 |
| Anti- mouse F4/80 (PerCP-Cy5.5) | Biolegend | 1:50 | 123128 |
| Anti- mouse ly6G (PE-Cy7) | Biolegend | 1:50 | 127618 |
| p-JAK2 | Cell Signaling Technology | 1:50 | 4406T |
| p-STAT3 | Cell Signaling Technology | 1:50 | 9145T |
| p-IκBα | Cell Signaling Technology | 1:50 | 2859S |
| P65 | Cell Signaling Technology | 1:50 | 8242S |
| Anti-mouse TNF-α (AF488) | BD biosciences | 1:100 | 557722 |
| Anti-mouse IL-1β (APC-ef780) | eBioscience | 1:100 | 47711482 |
| Anti-human CD3 (V450) | BD biosciences | 1:50 | 561812 |
| Anti-human TNF-α (PE) | BD biosciences | 1:50 | 554513 |
| Anti-human IFN-γ (APC) | BD biosciences | 1:50 | 554702 |
| Anti-human IL-2 (FITC) | BD biosciences | 1:50 | 554565 |
| Anti-human IL-8 (FITC) | Invitrogen | 1:50 | BMS136FI |
| Anti-human IL-11b (BV510) | BD biosciences | 1:50 | 563088 |
| Anti-human IL-14 (PerCP-Cy5.5) | Biolegend | 1:100 | 367110 |
| Anti-human IL-1β (AF647) | Biolegend | 1:100 | 511707 |

**Table S8. Secondary Reagents Used for Flow Cytometry**

|  | **Conjugate(s)** | **Source** | **Dilution** |
| --- | --- | --- | --- |
| Anti-rabbit | Alexa Fluor 488 | Thermo Fisher | 1:1000 |
| Anti-rabbit | Alexa Fluor 647 | Thermo Fisher | 1:1000 |

**Gating Strategy** (Figure 2i)


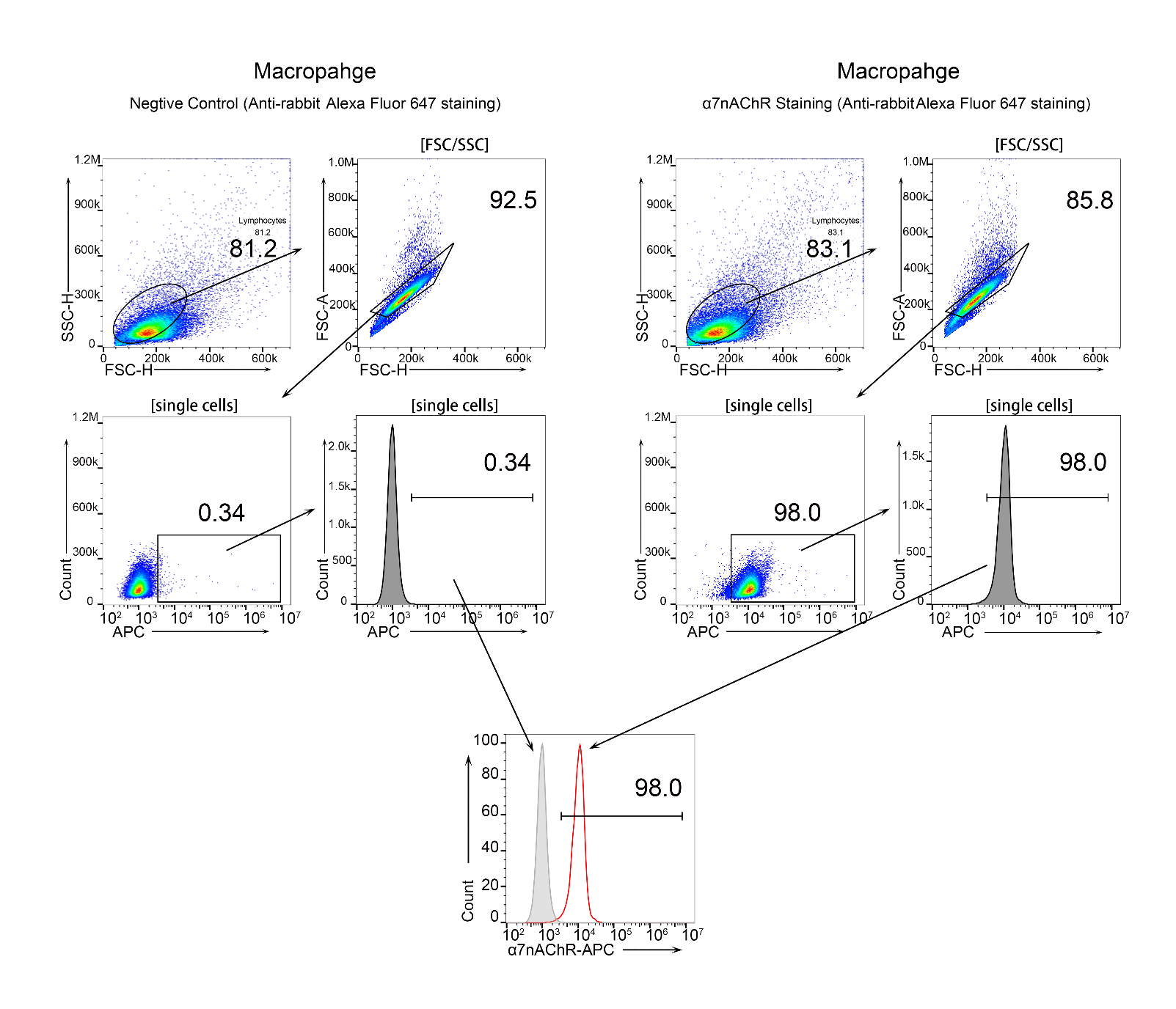

Supplement: Supplementary file 1 — supplemental material [file 41392_2022_1124_MOESM1_ESM.docx]
